# Exploration of effective biomarkers for venous thrombosis embolism in Behçet’s disease based on comprehensive bioinformatics analysis

**DOI:** 10.1038/s41598-024-66973-3

**Published:** 2024-07-10

**Authors:** Chunjiang Liu, Yuan Wang, Zhifeng Wu, Xiaoqi Tang, Guohua Wang, Jiajia Wang

**Affiliations:** 1 Division of Vascular Surgery, Department of General Surgery, Shaoxing People’s Hospital, Shaoxing, 312000 China; 2 Department of Intervention Vascular, Hefei Hospital of Anhui Medical University, Hefei, 230000 China; 3 Medical College, Shaoxing University, Shaoxing, 312000 China; 4 Department of Rheumatology, Shaoxing People’s Hospital, 568# Zhongxing North Road, Shaoxing, 312000 China

**Keywords:** Behçet’s disease, Venous thrombosis embolism, Diagnostic biomarker, Bioinformatics analysis, Machine learning, Computational biology and bioinformatics, Genetics, Immunology, Rheumatology, Cardiovascular diseases

## Abstract

Behçet’s disease (BD) is a multifaceted autoimmune disorder affecting multiple organ systems. Vascular complications, such as venous thromboembolism (VTE), are highly prevalent, affecting around 50% of individuals diagnosed with BD. This study aimed to identify potential biomarkers for VTE in BD patients. Three microarray datasets (GSE209567, GSE48000, GSE19151) were retrieved for analysis. Differentially expressed genes (DEGs) associated with VTE in BD were identified using the Limma package and weighted gene co-expression network analysis (WGCNA). Subsequently, potential diagnostic genes were explored through protein–protein interaction (PPI) network analysis and machine learning algorithms. A receiver operating characteristic (ROC) curve and a nomogram were constructed to evaluate the diagnostic performance for VTE in BD patients. Furthermore, immune cell infiltration analyses and single-sample gene set enrichment analysis (ssGSEA) were performed to investigate potential underlying mechanisms. Finally, the efficacy of listed drugs was assessed based on the identified signature genes. The limma package and WGCNA identified 117 DEGs related to VTE in BD. A PPI network analysis then selected 23 candidate hub genes. Four DEGs (*E2F1*, *GATA3*, *HDAC5*, and *MSH2*) were identified by intersecting gene sets from three machine learning algorithms. ROC analysis and nomogram construction demonstrated high diagnostic accuracy for these four genes (AUC: 0.816, 95% CI: 0.723–0.909). Immune cell infiltration analysis revealed a positive correlation between dysregulated immune cells and the four hub genes. ssGSEA provided insights into potential mechanisms underlying VTE development and progression in BD patients. Additionally, therapeutic agent screening identified potential drugs targeting the four hub genes. This study employed a systematic approach to identify four potential hub genes (*E2F1*, *GATA3*, *HDAC5*, and *MSH2*) and construct a nomogram for VTE diagnosis in BD. Immune cell infiltration analysis revealed dysregulation, suggesting potential macrophage involvement in VTE development. ssGSEA provided insights into potential mechanisms underlying BD-induced VTE, and potential therapeutic agents were identified.

## Introduction

Behçet’s disease (BD) is a chronic autoimmune disorder characterized by widespread vasculitis that can affect any bodily system^1^. While the prognosis for most BD patients is favorable, cardiovascular involvement significantly worsens the outlook. It is now understood that the mortality rate is directly correlated with cardiovascular system involvement, with venous thromboembolism (VTE) occurring frequently in approximately 50% of BD cases^2^. Inflammation-driven endothelial cell dysfunction is thought to be the main cause of thromboembolism in patients with BD^3^. In the absence of traditional cardiovascular risk factors, compromised immune-inflammatory responses, along with neutrophil activation, endothelial dysfunction, and coagulation abnormalities, can directly contribute to thrombus formation in BD patients^4,5^. Current evidence suggests that the likelihood of venous thrombosis is particularly heightened during the active phase of the disease, likely due to the complex coagulation mechanisms activated during this period^6^.

VTE, encompassing deep vein thrombosis (DVT) and pulmonary embolism (PE), represents the third most common cardiovascular disease after acute coronary heart disease and stroke^7^. This global health concern is responsible for a significant number of deaths annually and imposes substantial economic and psychological burdens on individuals and society^8^. Notably, over 50% of VTE patients lack clear symptoms and signs, leading to a high rate of misdiagnosis in clinical practice^9^. The resemblance of VTE symptoms to common conditions like dyspnea or post-exertional discomfort further complicates diagnosis. Pulmonary embolism, a potentially fatal complication in BD patients, can manifest with non-specific symptoms like chest pain and breathing difficulties. In some cases, extensive PE may even be the sole presenting feature of sudden death^10^. Therefore, prompt detection and treatment of VTE in BD are crucial for preventing VTE-related complications and mortality. Biomarkers offer significant potential for early identification and intervention in conditions like BD and asymptomatic VTE, which often present with subtle clinical manifestations. Timely diagnosis and treatment of VTE in BD patients is essential for mitigating adverse outcomes and improving patient prognosis.

Despite extensive global research on the pathogenesis of venous thrombosis in BD, the specific mechanisms underlying its occurrence remain elusive^4^. Microarray-based gene expression profiling has emerged as a prominent tool in biomedical research, enabling the identification of potential biomarkers. Studies have identified candidate biomarkers for both BD and VTE, including *IL10, FCRL3, MASP1, NF2, FAM3B,* and *MGMT* for BD^11^, and *ISG15*, *RPS15A*, *MRPL13*, *ICT1*, *MRPL15*, and *RPLP0* for VTE^12^. Additionally, research suggests that circulating microRNAs (miR-20a-3p, miR-134, miR-522-3p, and miR-874) may regulate genes encoding exosome components (*TLN1*, *LAMTOR3*, *STX7*, and *IMPA1*), potentially influencing exosome involvement in VTE pathophysiology^13^. While the precise etiology of BD remains unknown, it is likely a complex interplay of factors, including infections, genetics, epigenetics, and immunological components^14^. Emerging evidence points towards a growing link between venous thrombosis and the immune system, with inflammatory factors playing a confirmed role. The intricate interplay between enzymes and cellular processes in the endothelium, platelets, and white blood cells can create an inflammatory state, ultimately leading to acute vein thrombosis^15,16^. Biomarkers related to immune function may, therefore, hold promise as valuable predictors of VTE susceptibility in BD patients and could aid in treatment decisions. However, the literature on the specific genetic mechanisms underlying BD-induced VTE is scarce, highlighting the need for further investigation.

Bioinformatics techniques have revolutionized disease research by facilitating the exploration of underlying mechanisms and the identification of potential biomarkers. Extensive utilization of various analytical approaches, including Limma analysis, weighted gene co-expression network analysis (WGCNA), protein–protein interaction (PPI) analysis, machine learning algorithms, and nomogram evaluation, can lead to a deeper understanding of diseases and offer novel insights and approaches for disease prevention, early diagnosis, and treatment^17,18^. The advent of high-throughput technologies has significantly expanded the application of genomic data in biomarker discovery. However, a common challenge arises when the number of features in biomedical data exceeds the number of samples, potentially hindering the reproducibility of traditional feature selection methods^19,20^. Machine learning algorithms, such as Support Vector Machine-Recursive Feature Elimination (SVM-RFE), Least Absolute Shrinkage and Selection Operator (LASSO), and Random Forest, have emerged as crucial tools for identifying promising biomarkers. These methodologies expedite the exploration of underlying biological processes and aid in the identification of reliable biomarkers. Recent studies suggest that machine learning techniques, known for their predictive flexibility, outperform traditional regression methods in terms of accuracy^21^. This study addresses a critical gap in our understanding of the genetic mechanisms underlying BD-induced VTE. We employed a combination of bioinformatics and machine learning methodologies to uncover potential disease mechanisms and identify potential biomarkers.

## Methods

### Microarray data

All gene expression datasets employed in this analysis are publicly available through the National Center for Biotechnology Information (NCBI) Gene Expression Omnibus (GEO) database (https://www.ncbi.nlm.nih.gov/geo/). Dataset GSE209567 encompasses gene expression data from 29 BD patients and 15 healthy controls. Additionally, datasets GSE48000 (comprising 107 VTE patients and 25 healthy controls) and GSE19151 (including 70 VTE patients and 63 healthy controls) encompass data from VTE patients and their corresponding controls. The genes within these datasets were converted into probes and linked to their corresponding annotation symbols. Table 1 provides a comprehensive overview of the datasets used in this study.

**Table 1:** Detailed information of the GEO datasets in the study.

ID	GSE series	Disease	Samples	Source types	Platform	Group
1	GSE209567	BD	29 BD patients and 15 normal controls	Peripheral blood	GPL570	Discovery cohort
2	GSE48000	VTE	107 VTE patients and 25 normal controls	Peripheral blood	GPL10558	Discovery cohort
3	GSE19151	VTE	70 VTE patients and 63 normal controls	Peripheral blood	GPL571	Validation cohort

### Data processing and differential expression analysis

Preprocessing and standardization are essential steps for ensuring the quality and reliability of microarray data. These procedures eliminate biases and inconsistencies inherent to raw biological data, promoting data integrity. In this study, we employed various techniques, including background correction, normalization, and probe data visualization. The “afy” package in R was used to implement these methods. Additionally, the “surrogate variable analysis” package in R was utilized to mitigate the confounding effects of batch variations. Limma^22^, a popular R package for differential gene expression analysis, was employed to identify differentially expressed genes (DEGs) between the BD and control groups in the GSE209567 dataset. Likewise, the Limma package was used to identify DEGs between the VTE and control groups within the GSE48000 dataset. Specifically, utilizing the obtained expression profile dataset, the lmFit function was applied to perform multiple linear regression. The eBayes function was then employed to compute modeled t-statistics, moderated F-statistics, and log-odds of differential expression, with empirical Bayes moderation of the standard errors towards a common value, to ascertain the significance of gene expression differences. Additionally, heat maps and volcano plots for DEGs were generated using the ggplot2 R package. A false discovery rate (FDR) below 0.05 and a fold change (FC) exceeding 1.2 were set as the criteria for selecting DEGs.

### Weighted gene co-expression network analysis (WGCNA)

WGCNA, a widely employed technique, was utilized to construct co-expression networks and identify modules of genes exhibiting strong correlations with BD^23^. The analysis commenced with the calculation of the median absolute deviation (MAD) for each gene. Subsequently, the 50% of genes with the lowest MAD values were excluded. To construct a scale-free co-expression network, the expression matrix of DEGs was filtered using the goodSamplesGenes function within WGCNA. The pick-Soft-Threshold function was then employed to determine the adjacency between genes. This function utilizes co-expression similarity to compute the soft-thresholding power (β). The resulting adjacency relationships were transformed into a topological overlap matrix (TOM). Subsequently, the gene dissimilarity and its corresponding ratio were calculated. Hierarchical clustering and dynamic tree-cutting algorithms were then implemented to identify gene modules. Finally, a truncation value was chosen for the module tree diagram. By merging specific modules based on the dissimilarity of the estimated module feature genes, the co-expression network of these feature genes was visualized.

### Functional enrichment analysis

Gene Ontology (GO) enrichment analysis was employed to investigate the functional roles of DEGs in BD and VTE. This analysis aimed to identify enriched biological processes (BP), molecular functions (MF), and cellular components (CC) associated with the DEGs. Furthermore, the Kyoto Encyclopedia of Genes and Genomes (KEGG) pathway analysis was performed to explore enriched pathways potentially involved in BD and VTE^24^. To achieve comprehensive functional enrichment analysis, we utilized the clusterProfiler package in R, integrating both GO and KEGG analyses^25^. For visualization purposes, the top 10 enriched GO terms in each category were selected based on a significance threshold of FDR < 0.05 and p-value < 0.05.

### Protein–protein interaction network construction

The Search Tool for the Retrieval of Interacting Genes (STRING) database (version 12.0) (http://string-db.org) was employed to construct a PPI network of DEGs. The STRING database provides interaction scores, with interactions exceeding a combined score of 0.4 considered significant for this analysis. Cytoscape software (version 3.9.1) was then utilized to visualize the interactive network data. Furthermore, to identify hub genes within the network, the CytoHubba plug-in for Cytoscape was employed. Three algorithms (Betweenness Centrality, Closeness Centrality, and Degree Centrality) were implemented within CytoHubba. Each algorithm assigned a score to each DEG based on its network properties, enabling subsequent ranking of the genes according to their respective scores. The top 30 DEGs identified by each algorithm were designated as node DEGs. Finally, a Venn diagram was constructed to visually represent the overlap of node DEGs identified by all three algorithms, facilitating further analysis.

### Machine learning algorithms

To identify potential biomarkers for VTE in BD patients, three machine learning algorithms were employed: LASSO regression, SVM-RFE, and Random Forest. LASSO regression analysis was implemented to mitigate potential overfitting by shrinking less relevant coefficients towards zero, effectively performing variable selection^26^. SVM-RFE is particularly well-suited for datasets with a limited number of samples as it iteratively eliminates redundant features while retaining only those most relevant for classification^27^. Random Forest analysis offers advantages in handling high-dimensional datasets, constructing robust predictive models, and assessing the variable importance^28^. The intersection of genes identified by all three algorithms was considered a set of putative biomarkers for further investigation.

### Evaluation of receiver operating characteristic curve (ROC) and nomogram

The expression levels of candidate genes were compared between the VTE and control groups, as well as between the BD and control groups, using Student's t-test. This analysis aimed to identify statistically significant differences in gene expression between the groups. The diagnostic performance of candidate gene expression levels was subsequently evaluated using ROC curves. The area under the curve (AUC) was calculated using the “pROC” package in R to assess the discriminatory power of each gene^29^. To develop a multi-gene prognostic model for predicting VTE incidence in BD patients, a nomogram was constructed using the R package “RMS”^30^. This model integrates risk scores derived from the expression levels of the identified genes. The accuracy of the nomogram model was further validated using ROC curves.

### qRT-PCR of the hub genes and evaluation of the predictive model

To validate the identified hub genes in vivo, a retrospective study was conducted at Shaoxing People's Hospital. Peripheral blood samples were collected from patients diagnosed with BD (n = 8) and those diagnosed with both BD and VTE (n = 6) between June 1, 2022, and June 1, 2024. All diagnoses of BD adhered to the established International Criteria^31^. VTE was confirmed through duplex sonography of the lower limbs or computed tomographic pulmonary angiography (CTPA). Detailed clinical characteristics of these patients are presented in Supplementary Table S1. This study was performed in accordance with the ethical standards of the Declaration of Helsinki. The hospital's Ethics Committee approved the specimen collection protocol (Approval number: IEC-K-AF-016-1.2), and informed consent was obtained from all patients. Following collection, total RNA was extracted from peripheral blood using the MolPure® Blood RNA Kit according to the manufacturer’s instructions. RNA concentration was determined using a Nanodrop instrument (ThermoFisher, USA). cDNA synthesis was subsequently performed using the Hifair® II 1st Strand cDNA Synthesis Kit (11121ES60, Yeasen, Shanghai, China). The specific primers utilized in this analysis are listed in Supplementary Table S2. The expression levels of the hub genes were then compared between the two patient groups. Furthermore, a predictive nomogram was developed to discriminate between BD patients with VTE and those without VTE.

### Immune infiltration analysis

The cellular composition of infiltrating immune cells within the tissue samples was deconvoluted from the normalized gene expression matrix using the CIBERSORT algorithm^32^. This algorithm estimates the relative abundances of 22 distinct immune cell types within the samples. The R package “CIBERSORT” was employed to quantify the proportions of these immune cell types between the VTE and control groups. To visualize potential correlations between different immune cell populations in the context of VTE development, a heatmap was generated using the R package “corrplot”. Furthermore, single-sample gene set enrichment analysis (ssGSEA) was performed to investigate the associations between immune cell infiltration and characteristic gene expression profiles. The “ggcorrplot” package was utilized to generate graphical representations of these associations.

### ssGSEA and therapeutic agents screening

To elucidate potential connections between the identified biomarkers and hallmark gene sets, we employed ssGSEA. The hallmark gene sets were obtained from the Molecular Signatures Database (MSigDB) (https://www.gsea-msigdb.org/gsea/msigdb/collections.jsp), a comprehensive resource encompassing 50 well-defined biological states and processes. We utilized the GSVA software to perform ssGSEA and evaluate the correlations between the potential biomarkers and these hallmark gene sets.

Following ssGSEA, we conducted an enrichment analysis using Enrichr (https://maayanlab.cloud/Enrichr/). This analysis, employing a significance threshold of p < 0.05, aimed to identify potential therapeutic agents that specifically interact with the hub genes.

### Statistical analysis

Statistical analyses were performed using R software (version 4.2.1) with relevant packages, GraphPad Prism (version 9.4.0), and SPSS (version 26.0) software packages. For continuous variables, comparisons between two groups were conducted using Student's t-test, with a p-value threshold of less than 0.05 considered statistically significant.

### Ethical approval statement

This study was performed in accordance with the ethical standards of the Declaration of Helsinki. The specimen collection protocol was approved by the Ethics Committee of Shaoxing People’s Hospital (Approval number: IEC-K-AF-016-1.2), and informed consent was obtained from all patients.

## Results

### Identification of DEGs for BD and VTE using Limma

**Figure 1:**
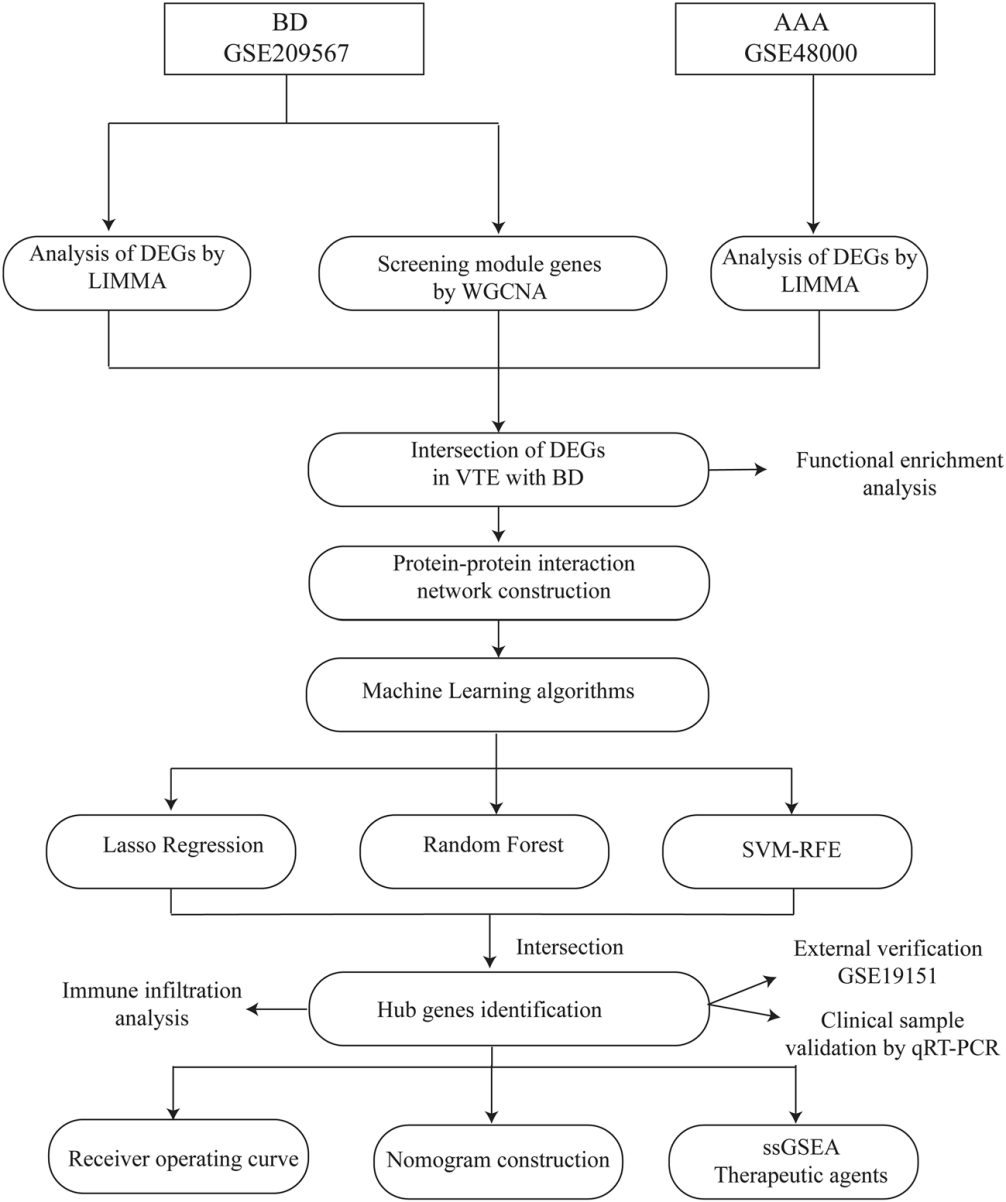
Flowchart of study.

**Figure 2:**
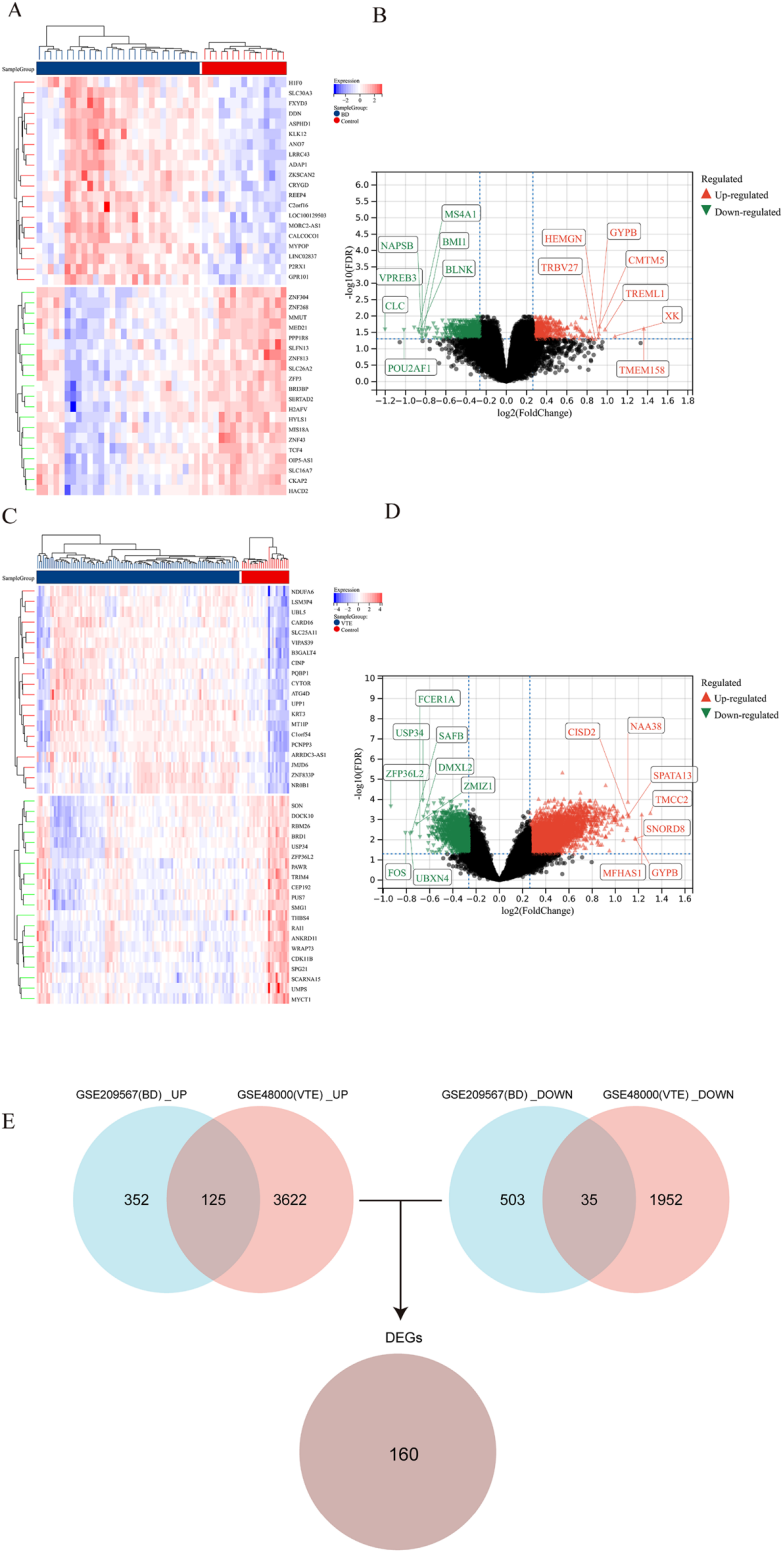
Heatmap and volcano plot of DEGs between BD and the control group, as well as between VTE and the control group. (**A**, **B**) Heatmap displaying the expression of the 20 most upregulated and downregulated DEGs. The red blocks represent upregulation, indicating increased gene expression in BD, while the blue blocks indicate downregulation, reflecting decreased gene expression. Volcano plot presenting all DEGs, with significantly upregulated genes labeled in red and downregulated genes labeled in green. (**C**, **D**) Heatmap and volcano plot of DEGs in VTE. The description remains consistent with the previous depiction. (**E**) The intersection of BD-related and VTE-related DEGs led to 160 DEGs.

Figure 1 illustrates the study flowchart. A total of 1015 genes exhibiting differential expression were identified in the comparison between the BD and control groups. Among them, 477 genes showed up-regulation, while 538 genes displayed down-regulation. Likewise, the comparison between the VTE and control groups revealed a total of 5734 genes exhibiting differential expression. Among these, 3747 genes were up-regulated, and 1987 genes were down-regulated. To visually represent these findings, a heatmap was generated to display the top 20 significantly differentially expressed genes in both up-regulated and down-regulated regulation (BD: Fig. 2A, VTE: Fig. 2C). Additionally, a volcano plot was utilized to illustrate the differentially expressed genes (BD: Fig. 2B, VTE: Fig. 2D). A total of 160 genes (Fig. 2E) were found to be overlapped between BD-related and VTE-related DEGs.

### Identification of significant module genes in BD via WGCNA

**Figure 3:**
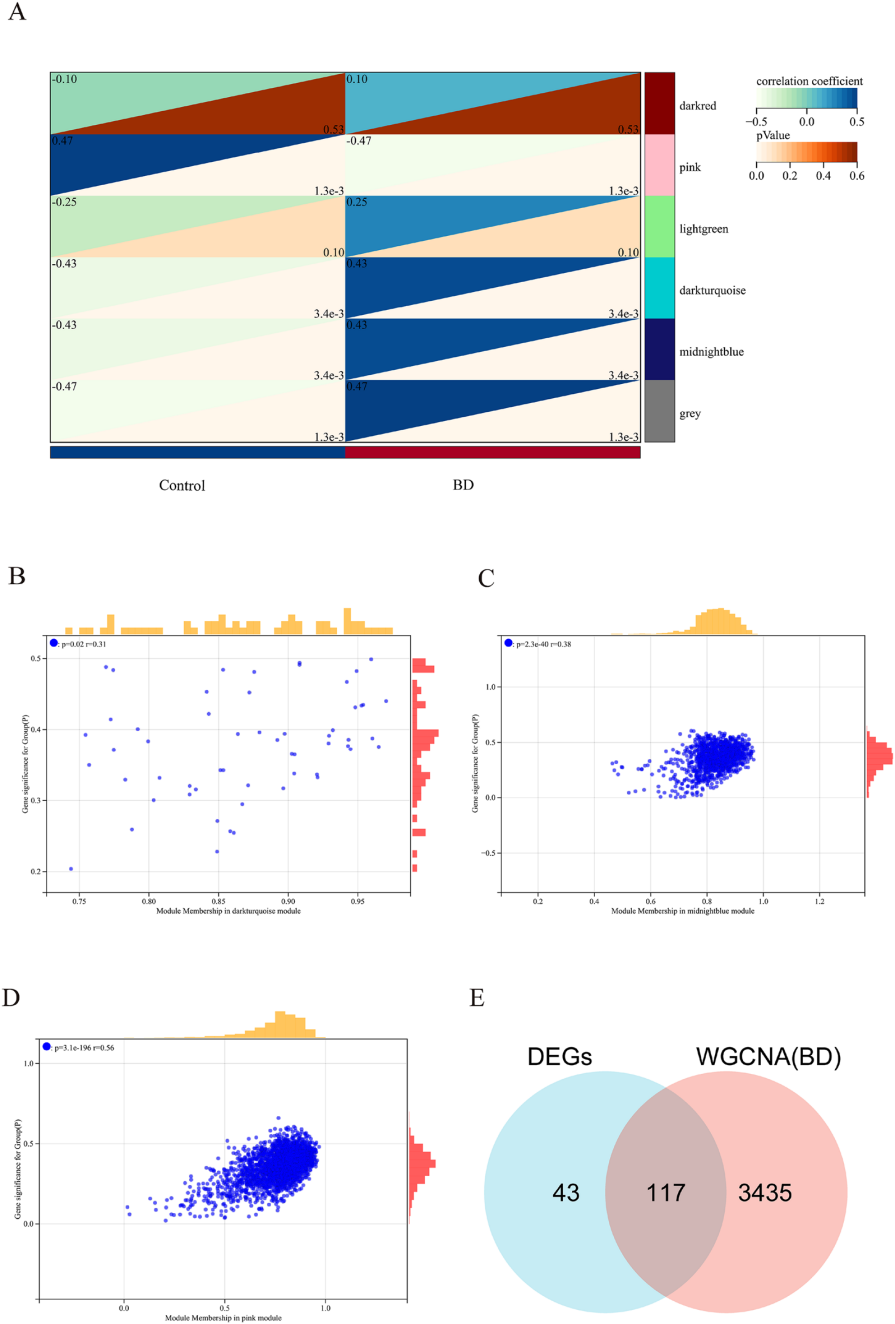
The recognition of module genes using WGCNA in BD. (**A**) Heatmap displays modules that are associated with BD. The correlation values between each module and BD are presented in the top left corner of the heatmap, while the p-values in the lower right corner indicate the statistical significance of these correlations. Among the modules within BD, the darkturquoise, midnightblue, and pink modules exhibit the strongest correlation. (**B**–**D**) The relationship between module membership and gene significance in the darkturquoise/midnightblue/pink modules of BD. (**E**) Venn plot illustrating the overlap of 160 DEGs and 3552 genes associated with BD modules, resulting in 117 DEGs associated with VTE in BD.

Significant module genes associated with BD were identified using WGCNA. The gray module, commonly referred to as the “junk module”, failed to cluster genes considered irrelevant or uninformative. The darkturquoise (r = 0.43, p = 3.4 × 10^–3^), midnightblue (r = 0.43, p = 3.4 × 10^–3^), and pink (r = − 0.47, p = 1.3 × 10^–3^) modules showed the highest correlation with BD (Fig. 3A). Figure 3B–D depict the relationship between module membership and gene significance in the darkturquoise/midnightblue/pink modules of BD. A total of 3552 genes were identified to be associated with BD. The intersection of these genes with the 160 DEGs yielded 117 DEGs associated with VTE in BD (Fig. 3E). Supplementary Fig. S1 displayed the selection of the soft threshold and the gene cluster tree.

### Functional enrichment analysis

**Figure 4:**
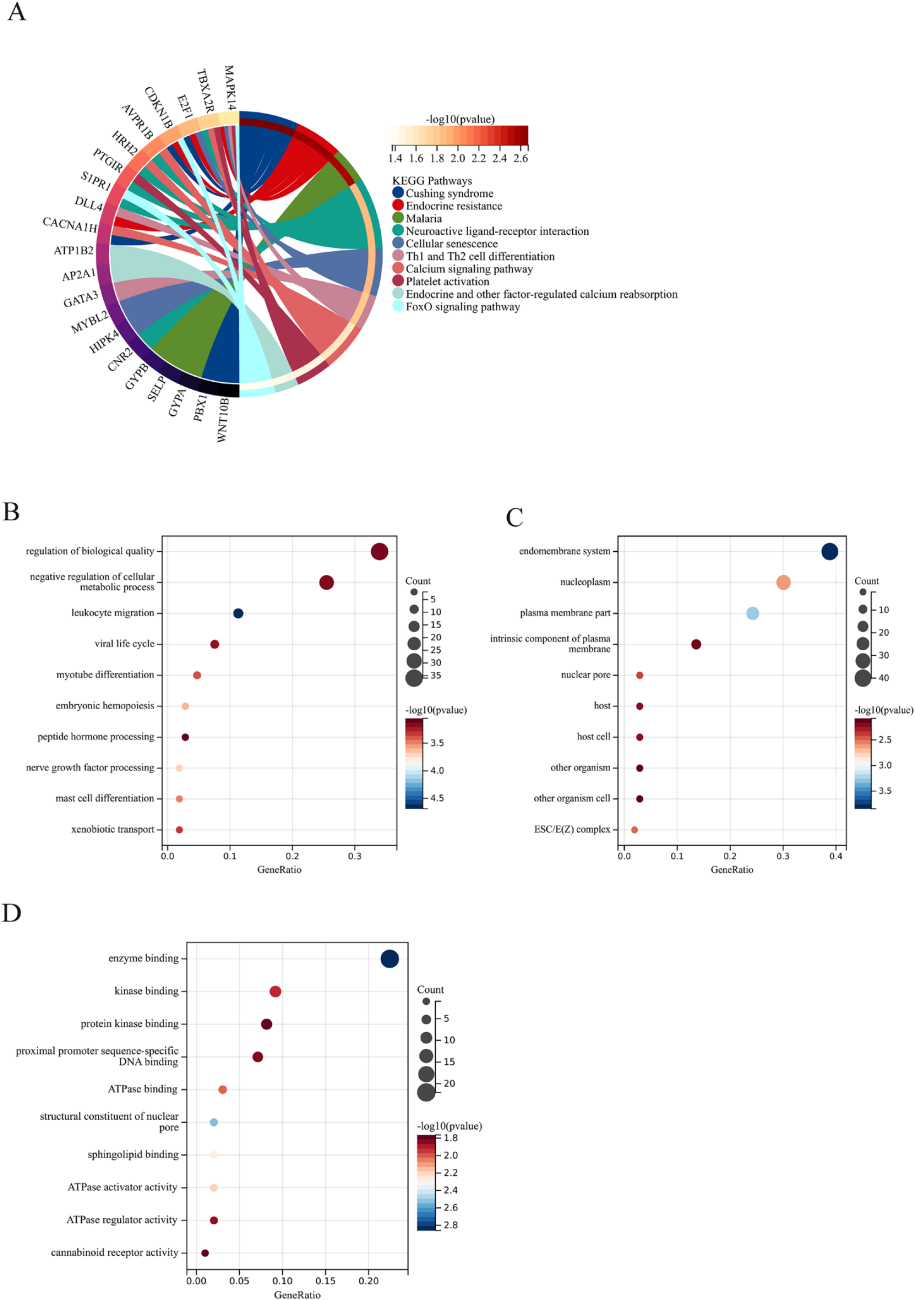
Functional enrichment analysis of DEGs associated with VTE in BD. (**A**) KEGG pathway analysis of DEGs associated with VTE in BD. (**B**–**D**) GO analysis (BP, CC, and MF) of DEGs associated with VTE in BD. The size of the circles corresponds to the number of genes, and the color indicates the significance level.

The analysis of functional pathways in the 117 DEGs (Fig. 4A, Supplementary Table S3) identified significant enrichment in various pathways, such as “Cellular senescence”, “Th1 and Th2 cell differentiation”, and “Platelet activation”. The BP analysis of the 117 DEGs demonstrated significant enrichment in GO terms, including “Negative regulation of cellular metabolic process”, “Leukocyte migration”, and “Regulation of biological quality”. Additionally, the CC analysis revealed enrichment in terms such as “Endomembrane system”, “Nucleoplasm”, and “Plasma membrane part”. MF analysis of the DEGs demonstrated significant associations with terms like “Enzyme binding”, “Kinase binding”, and “Protein kinase binding” (Fig. 4B–D, Supplementary Table S3).

### PPI network construction

**Figure 5:**
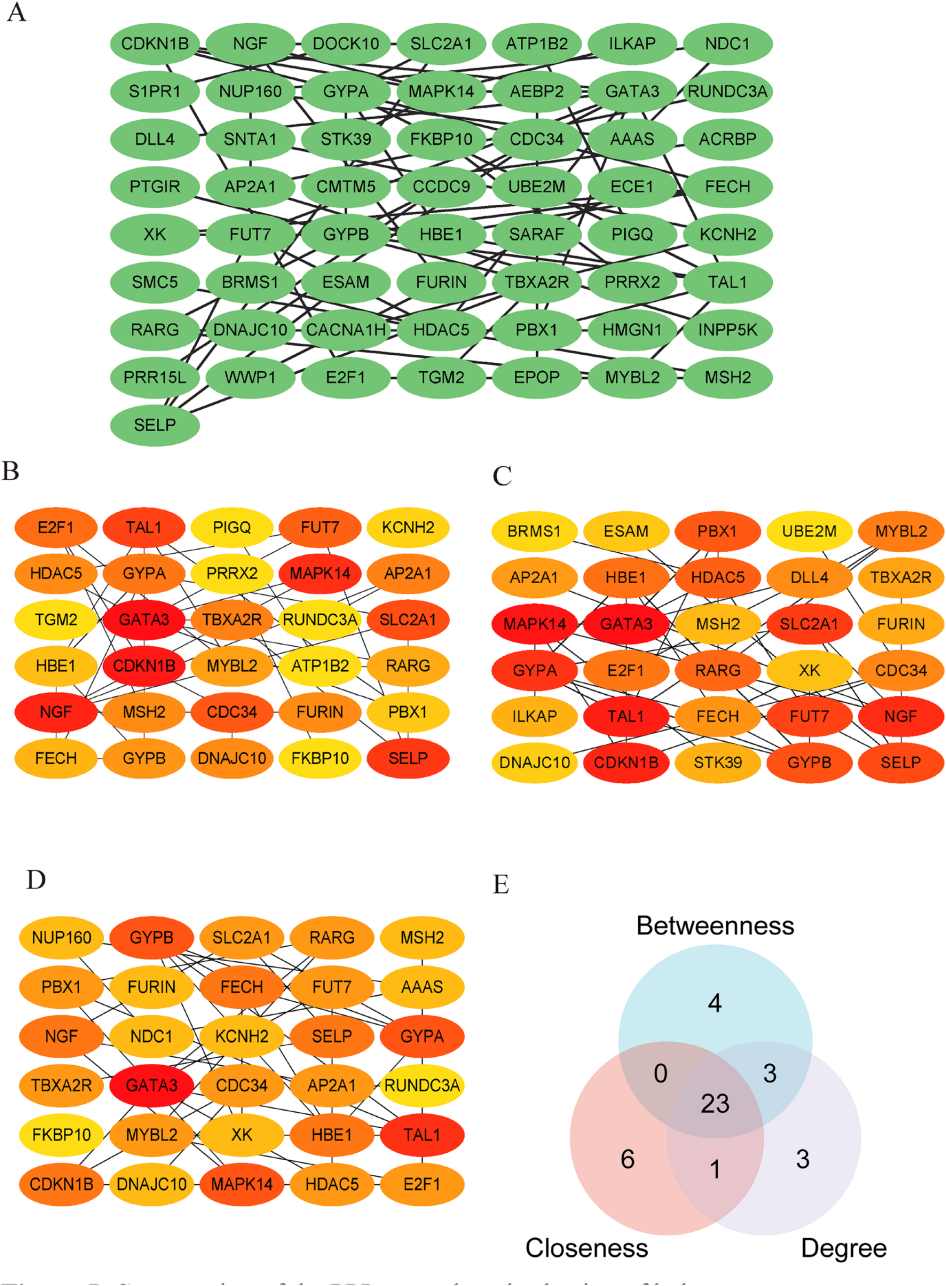
Construction of the PPI network and selection of hub genes. (**A**) Excluding 60 DEGs due to a lack of interaction, the preliminary PPI network was constructed using the remaining 57 DEGs. (**B**–**D**) Within the CytoHubba plug-in of Cytoscape, three distinct algorithms were utilized to select the top 30 DEGs. Panels (**B**–**D**) represent the application of betweenness, closeness, and degree algorithms, with darker colors indicating greater significance. (**E**) Through the intersection of genes identified by all three algorithms, a total of 23 DEGs were chosen.

A preliminary PPI network was constructed using the 117 DEGs to identify hub DEGs associated with BD and VTE. Among the DEGs, 57 genes were retained based on their interactions with other genes (Fig. 5A), while 60 DEGs lacking interaction were excluded. Furthermore, the CytoHubba plug-in was utilized, employing three distinct algorithms (degree, betweenness, and closeness) to identify the intersecting DEGs. The top 30 node genes determined by the betweenness, closeness, and degree algorithms are presented in Fig. 5B–D. The overlap of 30 genes identified by the three algorithms was visually represented in Fig. 5E, with 23 of these genes being identified as potential hub genes. Supplementary Table S4 provided comprehensive information on these 23 identified genes.

### Machine learning

**Figure 6:**
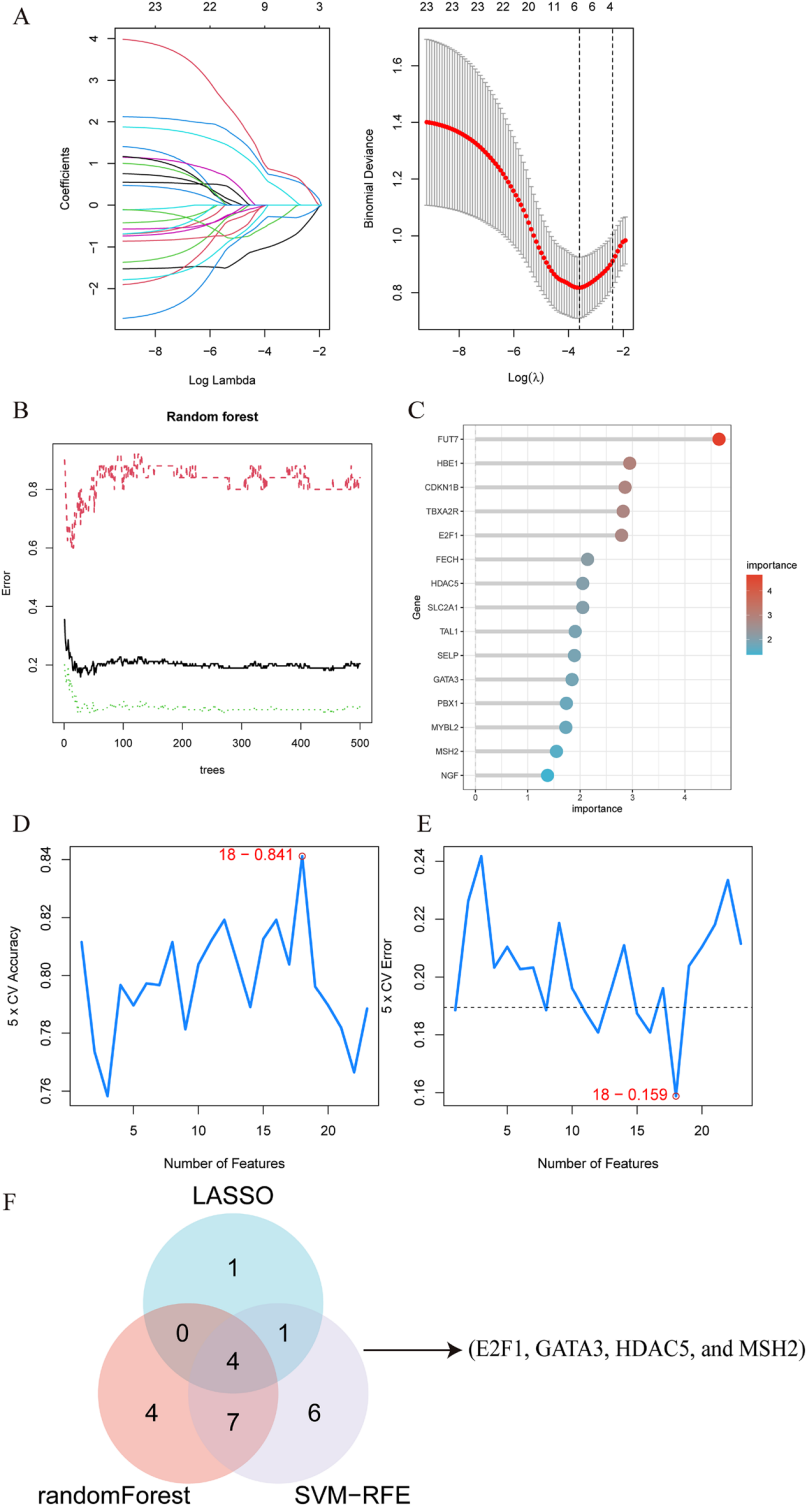
Identification of candidate diagnostic biomarkers through machine learning algorithms. (**A**) The Lasso regression algorithm identified six highly accurate biomarkers for diagnosing VTE with BD by precisely locating the curve's lowest point. (**B**, C) The random forest algorithm was employed to assess the error in VTE, leading to the ranking of the top 15 genes based on their importance scores. (**D**, **E**) The SVM-RFE algorithm selected 18 genes with the lowest error and highest accuracy. (**F**) Four hub genes (E2F1, GATA3, HDAC5, and MSH2) were identified as candidate biomarkers based on the intersection of the results from the three algorithms.

The LASSO regression algorithm was used to identify 6 candidate hub genes that serve as optimal biomarkers for diagnosing VTE in patients with BD. These genes correspond to the lowest point on the curve, as depicted in Fig. 6A. The random forest approach was utilized to evaluate the significance of DEGs, which successfully detected errors, as displayed in Fig. 6B. Figure 6C displays a compilation of the 15 most significant DEGs. Using SVM-RFE analysis, the top 18 DEGs with the lowest error and highest accuracy in diagnosing VTE in patients with BD were identified, as shown in Fig. 6D, E. Finally, four candidate hub genes (*E2F1*, *GATA3*, *HDAC5*, and *MSH2*) were selected by intersecting the genes identified through the three algorithms, as depicted in Fig. 6F.

### ROC, nomogram construction, and validation of the hub genes

**Figure 7:**
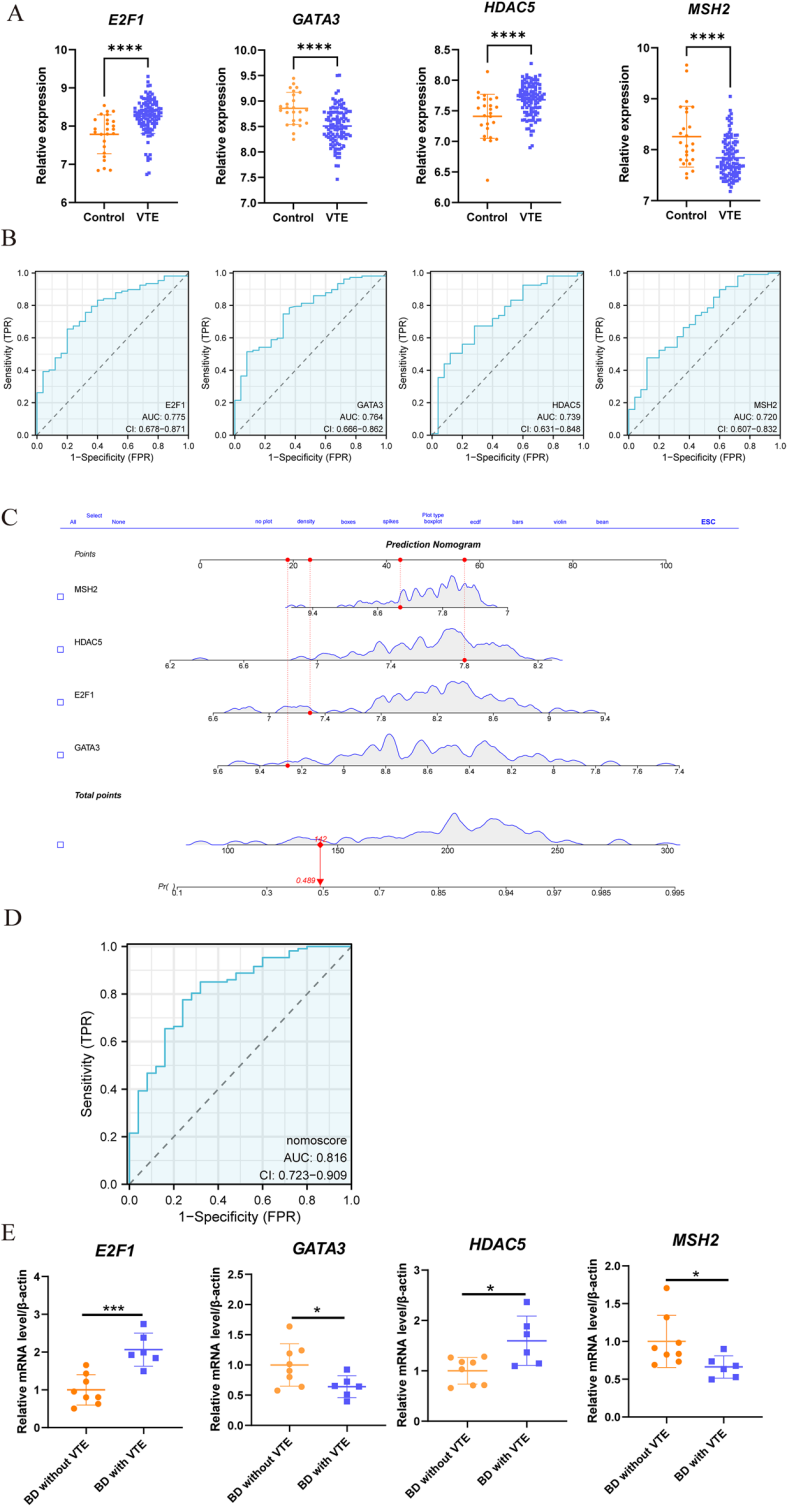
Evaluation of ROC and nomogram construction. (**A**) Differential expression is observed in four genes when comparing the VTE and control groups (****, p < 0.0001). (**B**) The ROC curve was utilized to evaluate the diagnostic efficacy of the four hub genes (E2F1, GATA3, HDAC5, and MSH2) in identifying VTE in patients with BD, demonstrating satisfactory diagnostic performance with AUC and 95% CI displayed in each panel. (**C**) The construction of a diagnostic nomogram employing the four genes aims to enhance the diagnosis of VTE in patients with BD. (**D**) The ROC curve for the nomogram is depicted in patients with BD presenting VTE. (**E**) Differential expression is observed in four genes via qRT-PCR when comparing the BD with VTE group and BD without VTE group (*, p < 0.05; ***, p < 0.001).

In comparison to the control group, VTE demonstrated the up-regulation of genes *E2F1* and *HDAC5*, as well as the down-regulation of genes *GATA3* and *MSH2*, as indicated in Fig. 7A. Figure 7B presented the AUC and 95% CI for each gene: *E2F1* (AUC: 0.775, 95% CI: 0.678–0.871); *GATA3* (AUC: 0.764, 95% CI: 0.666–0.862); *HDAC5* (AUC: 0.739, 95% CI: 0.631–0.848); *MSH2* (AUC: 0.720, 95% CI: 0.607–0.832). The analysis of the ROC curves demonstrated that these four genes exhibited satisfactory diagnostic performance. Finally, the construction of the nomogram, as depicted in Fig. 7C, resulted in an AUC of 0.816 (95% CI: 0.723–0.909), indicating significant clinical diagnostic value, as demonstrated in Fig. 7D. Similarly, BD showed the up-regulation of genes *E2F1* and *HDAC5*, as well as the down-regulation of genes *GATA3* and *MSH2*, as shown in Supplementary Fig. S2A. Supplementary Fig. S2B presented the AUC and 95% CI for each gene. The analysis of the ROC curves also revealed satisfactory diagnostic performance for these four genes. Finally, the construction of the nomogram, as depicted in Supplementary Fig. S2C, resulted in an AUC of 0.823 (95% CI: 0.699–0.947), confirming significant clinical diagnostic value, as demonstrated in Supplementary Fig. S2D. To further validate its diagnostic potential, the validation dataset GSE19151 was utilized for ROC curve analysis. The analysis of the nomogram in the validation dataset showed an AUC of 0.902, confirming substantial clinical diagnostic value, as illustrated in Supplementary Fig. S2E. Additionally, as depicted in Fig. 7E, clinical validation of peripheral blood samples revealed an elevated expression of two DEGs (*E2F1* and *HDAC5*) in BD patients with coexisting VTE in contrast to the BD-only group. In contrast, the expression levels of two additional DEGs (*GATA3* and *MSH2*) were found to be lower under the same conditions. Moreover, to assist in the differentiation of BD patients with potential VTE, we established a predictive nomogram. This model, presented in Fig. S3A–C, achieved an AUC of 1, substantiating its significant predictive capability for VTE in individuals with BD.

### Immune cell infiltration analysis

**Figure 8:**
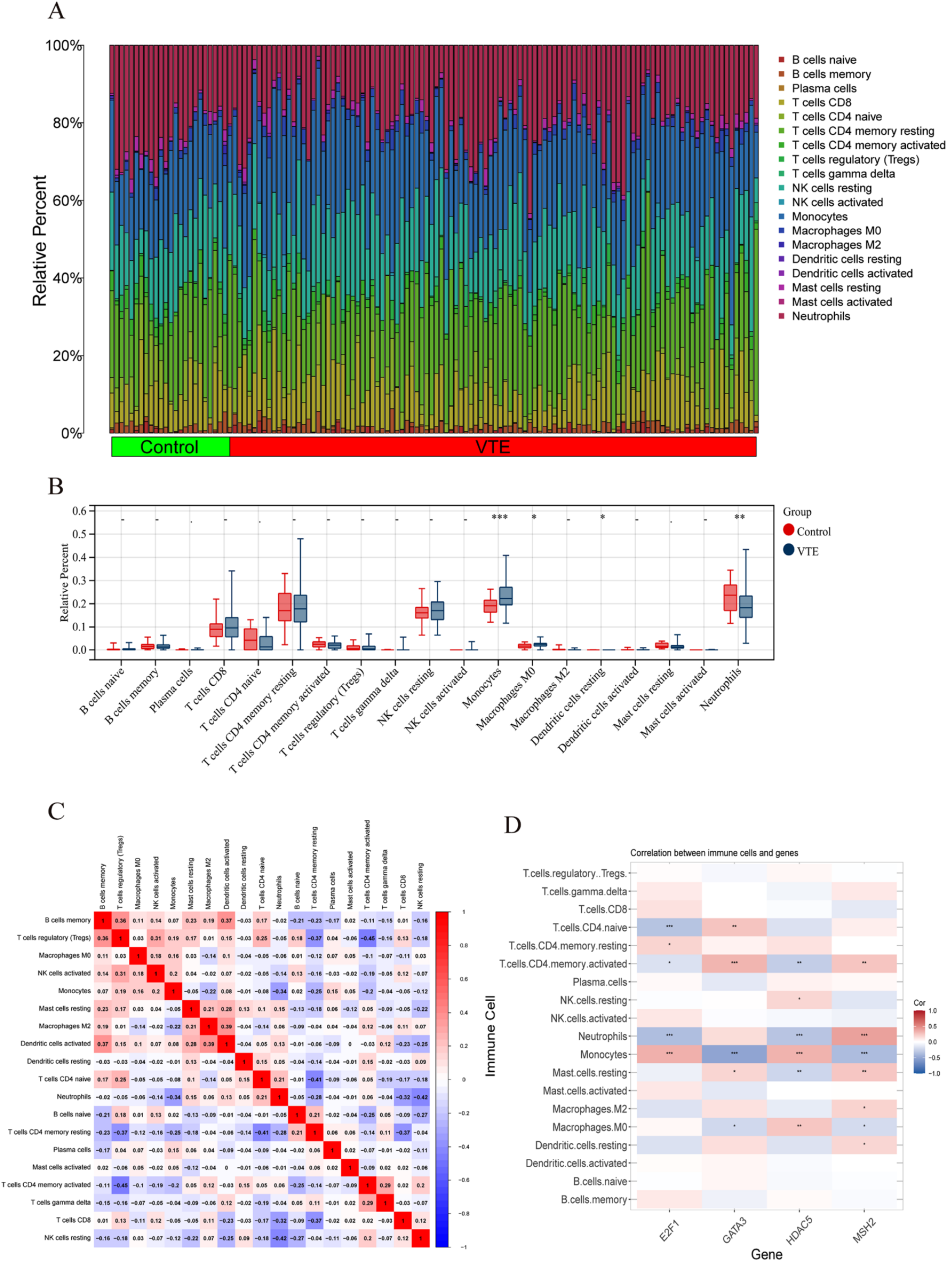
Immunological alterations between control and VTE groups, and correlations between hub DGEs and immunological features in VTE. (**A**) A bar plot representing the distribution of immune cells among different samples. (**B**) A boxplot comparing the expression of immune cells between the VTE and control groups, with statistical significance denoted as *p < 0.05, **p < 0.01, ***p < 0.001. (**C**) A heatmap illustrating the correlations among various immune cells implicated in the pathogenesis of VTE. (**D**) Correlation analysis assessing the relationship between immune cell infiltrations and the four identified hub DGEs.

Three distinct immune cell types exhibited zero percent distribution across all samples and were therefore excluded from further analysis. Figure 8A presents a bar plot depicting the distribution of the remaining 19 immune cell types following the application of the CIBERSORT algorithm. Boxplot analysis revealed a significantly higher prevalence of macrophages and M0 macrophages in the VTE group compared to the control group. Conversely, VTE samples displayed a lower prevalence of neutrophils (Fig. 8B). Correlation analysis identified a positive correlation between M2 macrophages and activated dendritic cells (r = 0.39). Regulatory T cells exhibited the strongest negative correlation with activated CD4 memory T cells (r = − 0.45), as shown in Fig. 8C. These findings suggest that modulating macrophage populations may hold promise as a therapeutic strategy for VTE. Additionally, analysis of immune cell infiltration demonstrated a significant correlation with all three identified hub DEGs, as illustrated in Fig. 8D.

### ssGSEA and therapeutic agent screening

Next, ssGSEA revealed significant associations between the four hub genes and diverse biological processes, including “hypoxia”, “heme metabolism”, “coagulation”, “cholesterol homeostasis”, and “angiogenesis”, as illustrated in Supplementary Fig. S4. These biological processes might be intricately connected to the onset and progression of VTE in individuals BD.

Enrichr database was utilized to screen therapeutic agents that target the four hub genes. The analysis of the predicted results indicated potential efficacy in targeting the four hub genes associated with VTE induced by BD. The identified therapeutic agents include chlorpromazine, dronabinol, trabectedin, vorinostat, and carmustine (Supplementary Fig. S5, Supplementary Table S5).

## Discussion

Recent research has increasingly highlighted the link between VTE and the immune system, with confirmed associations to inflammatory factors. DVT arises from complex interactions between enzymes and cellular processes. The endothelium, platelets, and white blood cells collaborate to induce a pro-inflammatory state, ultimately culminating in thrombosis and acute DVT^16,33^. VTE is associated with a significant mortality rate, particularly from PE. Untreated cases have a mortality rate of 30%. However, prompt diagnosis and treatment can reduce the mortality rate due to PE or treatment-related causes to less than 1%^34^. VTE also carries the burden of long-term complications and substantial healthcare costs. Approximately 50% of DVT patients may experience post-thrombotic syndrome, a chronic and debilitating condition. Additionally, the expensive treatment of VTE further burdens the healthcare system^35^. The presence of cardiovascular involvement worsens the prognosis of Budd-Chiari syndrome (BD). Therefore, considering these potential adverse consequences, early prevention, detection, and treatment of VTE are of paramount importance in patients with BD.

The precise mechanisms by which Budd-Chiari syndrome and VTE share transcriptional pathogenesis remain to be elucidated. To address this knowledge gap, we conducted a comprehensive analysis of microarray datasets, aiming to identify common biomarkers and biological pathways associated with the development of both diseases. Reliable biomarkers hold significant value in modern medicine. The application of bioinformatics and machine learning methodologies have significantly advanced the investigation of potential mechanisms and biomarker discovery. These methods offer a powerful approach for the accurate identification of disease-associated biomarkers, facilitating research into disease initiation, progression, and exploration of potential pathogenic mechanisms.

Assessment of hub gene expression levels in peripheral blood samples from patients diagnosed with BD and VTE offers valuable insights for predicting the risk of VTE development in this specific population. This approach, therefore, emerges as a potentially practical and efficacious clinical tool. Our nomogram facilitates the calculation of both cumulative and total scores for each gene. As a result, this nomogram holds significant promise for clinical application by aiding in the identification and early intervention of BD patients with elevated total scores. Early intervention has the potential to improve the prognosis of this patient population.

This study employed the Limma R package, a robust tool for gene expression data analysis, to identify DEGs associated with Budd-Chiari syndrome and VTE. Overlap analysis revealed 160 genes shared between BD- and VTE-associated DEGs. To further explore co-expressed genes potentially related to BD pathogenesis, a gene coexpression network was constructed using WGCNA. This analysis identified significant correlations between gene modules and phenotypes, leading to the discovery of 3552 genes associated with BD. Subsequent analysis of these genes alongside the 160 overlapping DEGs pinpointed 117 DEGs specifically associated with VTE in the context of BD. To elucidate the functional roles of these 117 DEGs, enrichment analyses were performed using the KEGG pathway and GO databases. The results indicated a primary association with cellular senescence, differentiation of Th1 and Th2 cells, platelet activation, negative regulation of cellular metabolism, and leukocyte migration. These findings suggest a strong link between immune system dysregulation, inflammatory responses, and the interplay between BD and VTE. Dysregulations in Th1/Th2 characterize BD's complex immune responses^36^. Th1 cell-driven inflammation can compromise vascular endothelium, elevating thrombosis likelihood. Additionally, cellular senescence is intimately linked with both BD and VTE^37,38^. The exacerbation of senescence-associated secretory phenotype (SASP) during cellular senescence contributes to platelet activation, a critical element in VTE development^39^. Moreover, leukocyte migration significantly influences the pathology of both BD and VTE. In BD sufferers, aberrant leukocyte migration and activation may intensify vascular inflammation and thrombosis risk^40^. These aspects support the view that there is a potential association between BD and VTE.

Furthermore, the 117 DEGs were mapped onto a PPI network to investigate potential interactions. This analysis identified 23 potential hub DEGs, which may play crucial roles in the underlying biological processes. Machine learning algorithms, including SVM-RFE, LASSO, and Random Forest, are increasingly employed for biomarker discovery due to their effectiveness in identifying promising candidates. These methodologies facilitate the exploration of complex biological processes and aid in the identification of reliable biomarkers. In this study, we utilized three machine learning methods alongside ROC curve analysis to evaluate the diagnostic accuracy of candidate gene expression. This approach led to the identification of four candidate hub genes with potential diagnostic value: *E2F1*, *GATA3*, *HDAC5*, and *MSH2*. To further validate the diagnostic potential of these genes, an additional dataset (GSE19151) and clinical validation using peripheral blood samples were employed. These analyses confirmed the significant clinical diagnostic value of the identified gene expression signature.

E2F1, the first identified member of the E2 promoter binding factor (E2F) family, is believed to play a critical role in cell cycle regulation, differentiation, apoptosis, and the DNA damage response^41^. Dysregulation of E2F1 can impact downstream transcriptional targets, leading to DNA replication stress. This stress arises when persistent obstacles, both internal and external, hinder DNA replication and ultimately contribute to various diseases, including cancer^42^. A study suggested a potential link between E2F1 and vascular diseases, as inhibiting its expression protected Human Umbilical Vein Endothelial Cells (HUVECs) from high-glucose-induced injury^41^. Overexpression of E2F1 has been demonstrably associated with the development and progression of various malignant tumors^43^. As established, active malignant tumors are a leading cause of VTE from an etiological perspective. Therefore, E2F1 may play a role, albeit potentially limited, in VTE pathogenesis^44^. VTE is a complex disease influenced by a multitude of factors, including environmental and genetic components. Numerous genes have been identified as conferring susceptibility to VTE. The established association between VTE and inflammation is further supported by the involvement of inflammatory cells like monocytes, platelet aggregation, and C-reactive protein^44^. Consequently, immune gene mutations linked to inflammation may contribute to VTE pathogenesis. Interestingly, E2F1 upregulation has been observed in both VTE and Behçet's disease, suggesting a potential connection between E2F1, VTE, and BD.

GATA3, a transcription factor belonging to the GATA family, plays a critical role in regulating cell differentiation and proliferation. Studies have shown a potential association between the immune response and low expression of GATA3 (Th2) in patients with Neuro-Behçet's disease (NBD)^45^. Interestingly, in active BD with skin lesions, the expression ratio of Th2/Th1 (GATA3/Tbet) in these lesions exhibits a tendency to increase. This suggests a potential bias towards the GATA3(Th2) axis during T cell immune activation within BD lesions^46^. These findings collectively indicate a close relationship between GATA3 and BD. Additionally, GATA3 has been demonstrated to possess tumor suppressive effects in various cancers, including osteosarcoma^47^. Nevertheless, the relationship between GATA3 and VTE remains uncertain.

Histone deacetylase 5 (HDAC5) is a member of the class IIa histone deacetylase family. Primarily localized within the nucleus of vascular smooth muscle cells (VSMCs), HDAC5 functions as a transcriptional repressor, suppressing the expression of its target genes. Upon phosphorylation, HDAC5 interacts with specific proteins, leading to the de-repression of genes associated with vascular diseases^48^. Interestingly, HDAC5 expression appears to be dynamically regulated in the endothelium. Studies suggest overexpression of *HDAC5* in endothelial cells during venous return, while normal endothelial venous flow conditions are associated with down-regulated *HDAC5* expression^49^. Additionally, *HDAC5* has been implicated in impaired angiogenesis, a hallmark of scleroderma (SSc). *HDAC5* inhibits pro-angiogenic factors in endothelial cells, contributing to the disease. Silencing *HDAC5* in SSc-affected endothelial cells restores normal angiogenic function^50^. This collective evidence strongly suggests a close link between *HDAC5* and vascular diseases. Additionally, research has shown that inhibiting *HDAC5* can enhance tissue-type plasminogen activator (t-PA) production, a molecule with antithrombotic properties, thereby ameliorating intravascular thrombosis^51^. While a study demonstrated that mutant forms of *HDAC5* (HDAC5AA) activate the mTOR pathway and promote the survival and regeneration of retinal ganglion cells (RGCs)^52^, it is important to note that BD can significantly affect patients' eyes, establishing a connection between BD and vascular-related diseases. However, the precise relationship between BD and VTE remains to be elucidated.

MutS homolog 2 (MSH2) is a well-established key component of the mismatch repair (MMR) system. The MMR system plays a critical role in maintaining genomic stability by recognizing and repairing mismatched nucleotides during DNA replication. Mutations in genes encoding components of the MMR system, including MSH2, have been implicated in the pathogenesis of various autoimmune and autoinflammatory diseases. These mutations may disrupt the tightly regulated activity of pattern recognition receptors (PRRs) and their signaling pathways^53^. Moreover, MSH2 has been demonstrably linked to the initiation and progression of cancer, potentially impacting tumorigenesis, development, and immune regulation^54^. However, current research on MSH2 function primarily focuses on specific cancer types, with limited investigation into its role in VTE and BD.

BD is a chronic, systemic, and inflammatory vascular disorder characterized by vasculitis and endothelial injury. While the precise etiology of VTE associated with BD remains to be fully elucidated, the leading hypothesis suggests a complex interplay between neutrophil activation, endothelial dysfunction, and coagulation abnormalities in the pathogenesis of BD-induced VTE^4,5^. Neutrophil activation leads to the release of high levels of neutrophil extracellular traps (NETs), which contribute to endothelial cell injury and alterations in coagulation function. Additionally, inflammation triggers the release of proinflammatory factors and chemokines, which in turn activate platelets, leukocytes, and endothelial cells. This activation cascade results in vascular endothelial dysfunction, ultimately promoting thrombosis. BD patients exhibit a pre-thrombotic state characterized by dysregulation of the hemostasis, coagulation, and anticoagulation systems. This dysregulation, potentially arising from alterations in the coagulation mechanism and platelet activation, ultimately contributes to the development of VTE in these patients. The significantly elevated risk of VTE in BD patients presents a substantial clinical challenge. A comprehensive understanding of the relationship between BD and VTE, alongside the identification of reliable disease markers, is crucial for facilitating early diagnosis and effective treatment, thereby improving the prognosis for BD patients.

Subsequent analysis of immune cell infiltration in VTE patients revealed a significantly higher prevalence of macrophages compared to the control group. Conversely, VTE samples displayed a lower prevalence of neutrophils. This finding appears to contradict previous literature reporting an increase in neutrophil numbers in VTE^55^. The observed discrepancy may be attributed to the inclusion of recurrent and chronic VTE samples in the utilized GEO dataset. Neutrophils undergo activation during the acute phase of DVT development; however, their numbers are likely to decline in the chronic phase due to depletion. Existing literature supports the significant role of macrophages in the pathogenesis and progression of both BD and VTE^56–58^. Inflammasome activation in macrophages leads to the release of tissue factor, a critical trigger for thrombosis^59^. Our findings regarding macrophage prevalence align with these previous studies. Consequently, we investigated the potential interrelationship between the four identified hub genes (*E2F1*, *GATA3*, *HDAC5*, and *MSH2*) and immune cell infiltration. This analysis revealed a positive correlation between immune-associated macrophages and the expression of all four hub genes (*E2F1*, *GATA3*, *HDAC5*, and *MSH2*). In conclusion, these findings suggest that modulating macrophage populations may present a promising therapeutic strategy for managing VTE in patients with BD.

The ssGSEA analysis revealed significant associations between the four identified hub genes and various biological processes, including hypoxia, heme metabolism, coagulation, cholesterol homeostasis, and angiogenesis. These findings suggest that the four hub genes may be intricately linked to the initiation and progression of VTE in BD patients. Current treatment strategies for VTE in BD primarily focus on immunosuppressants, with anticoagulation therapy potentially unnecessary for managing DVT in this context. However, the high 5-year recurrence rate of 36.5%^60^ necessitates the exploration of more effective therapeutic options. To address this need, we employed the Enrichr database to screen for potential therapeutic agents targeting the four hub genes. The analysis identified several candidate drugs, including chlorpromazine, dronabinol, trabectedin, vorinostat, and carmustine, which exhibited potential efficacy in targeting the hub genes associated with BD-induced VTE.

Our research elucidates potential disease mechanisms and identifies biomarkers related to VTE induced by BD, employing a synthesis of bioinformatics and machine learning methodologies. The nomogram model crafted in our study exhibits significant predictive value for VTE in BD patients. Additionally, this study provides some directions for future studies delving into the molecular mechanisms underlying the pathogenesis of VTE precipitated by BD. However, despite these advantages, the study has limitations. Firstly, despite utilizing validation datasets and clinical samples for diagnostic assessment, further experimental research is essential to validate and explore the underlying mechanisms of VTE induced by BD. Secondly, this study was based on bioinformatics analysis, and the verification of hub genes was constrained by relatively small validation samples due to sample acquisition challenges, so further validation using multicenter studies involving larger sample sizes should be performed. Thirdly, although predicted therapeutic agents have been screened, the absence of concrete experimental corroboration means that significant further research is required to evaluate the efficacy of drug treatments targeting these hub genes for BD with VTE patients.

## Conclusion

This study employed a systematic approach to identify four candidate hub genes (*E2F1*, *GATA3*, *HDAC5*, and *MSH2*). A nomogram was subsequently constructed to facilitate the diagnosis of VTE in Budd-Chiari syndrome patients. Furthermore, analysis of immune cell infiltration revealed dysregulation in their relative proportions, suggesting a potential role for macrophages in VTE development within the context of BD. Importantly, ssGSEA provided insights into potential underlying mechanisms associated with BD-induced VTE. Additionally, the study identified potential therapeutic agents for further investigation.

### Supplementary Information

Supplementary Information.

## Data Availability

The datasets used in this study can be easily accessed and downloaded from the publicly accessible GEO database. GSE209567: https://www.ncbi.nlm.nih.gov/geo/query/acc.cgi?acc=GSE209567 (GPL570[HG-U133_Plus_2] Affymetrix Human Genome U133 Plus 2.0 Array). GSE48000: https://www.ncbi.nlm.nih.gov/geo/query/acc.cgi?acc=GSE48000 (GPL10558 Illumina HumanHT-12 V4.0 expression beadchip). GSE19151: https://www.ncbi.nlm.nih.gov/geo/query/acc.cgi?acc=GSE19151 (GPL571[HG-U133A_2] Affymetrix Human Genome U133A 2.0 Array). The study’s codes have been uploaded to the GitHub repository (https://github.com/wangjiajia-one/bioinformatics-R-code-1.0-.git).

## References

[1] Rodolfi S, Nasone I, Folci M, Selmi C, Brunetta E (2022). Autoinflammatory manifestations in adult patients. Clin. Exp. Immunol..

[2] Davatchi F (2017). Behcet’s disease: Epidemiology, clinical manifestations, and diagnosis. Expert Rev. Clin. Immunol..

[3] Pamuk ON (2024). Behçet’s Syndrome. N. Engl. J. Med..

[4] Bettiol A (2023). Vascular Behçet syndrome: From pathogenesis to treatment. Nat. Rev. Rheumatol..

[5] Toledo-Samaniego N (2022). Arterial and venous involvement in Behçet's syndrome: A narrative review. J. Thromb. Thrombol..

[6] Fernández-Bello I, López-Longo FJ, Arias-Salgado EG, Jiménez-Yuste V, Butta NV (2013). Behçet's disease: New insight into the relationship between procoagulant state, endothelial activation/damage and disease activity. Orphanet. J. Rare Dis..

[7] Oleksiuk-Bójko M, Lisowska A (2023). Venous thromboembolism: Why is it still a significant health problem?. Adv. Med. Sci..

[8] Wang D (2020). Risk of venous thromboembolism in patients undergoing gastric cancer surgery: Protocol for a systematic review and meta-analysis. BMJ Open.

[9] Cunningham MS, Preston RJ, O'Donnell JS (2009). Does antithrombotic therapy improve survival in cancer patients?. Blood Rev..

[10] Goldberg JB (2023). Surgical management and mechanical circulatory support in high-risk pulmonary embolisms: Historical context, current status, and future directions: A scientific statement from the american heart association. Circulation.

[11] Liu H (2023). Identification of the immune-related biomarkers in Behcet’s disease by plasma proteomic analysis. Arthr. Res. Therapy.

[12] Lu S, Lijuan R, Tang QH, Liu QL, Xian-Lan Z (2021). Bioinformatics analysis and identification of genes and molecular pathways involved in venous thromboembolism (VTE). Ann. Vasc. Surg..

[13] Chen X, Cao J, Ge Z, Xia Z (2021). Correlation and integration of circulating miRNA and peripheral whole blood gene expression profiles in patients with venous thromboembolism. Bioengineered.

[14] Al-Araji A, Kidd DP (2009). Neuro-Behçet's disease: Epidemiology, clinical characteristics, and management. Lancet Neurol..

[15] Huang SL (2023). Recent advances on the molecular mechanism and clinical trials of venous thromboembolism. J. Inflamm. Res..

[16] Momi S (2022). Proline-rich tyrosine kinase Pyk2 regulates deep vein thrombosis. Haematologica.

[17] Yang Y (2024). Biomarkers prediction and immune landscape in ulcerative colitis: Findings based on bioinformatics and machine learning. Comput. Biol. Med..

[18] Xue A (2024). Study on the neuroprotective effect of Zhimu-Huangbo extract on mitochondrial dysfunction in HT22 cells induced by D-galactose by promoting mitochondrial autophagy. J. Ethnopharmacol..

[19] Sun TH, Wang CC, Wu YL, Hsu KC, Lee TH (2023). Machine learning approaches for biomarker discovery to predict large-artery atherosclerosis. Sci. Rep..

[20] Zhang WY (2023). Analysis and validation of diagnostic biomarkers and immune cell infiltration characteristics in pediatric sepsis by integrating bioinformatics and machine learning. World J. Pediatr..

[21] Xing L (2022). Exploration of biomarkers of psoriasis through combined multiomics analysis. Mediat. Inflamm..

[22] Ritchie ME (2015). limma powers differential expression analyses for RNA-sequencing and microarray studies. Nucleic Acids Res..

[23] Langfelder P, Horvath S (2008). WGCNA: An R package for weighted correlation network analysis. BMC Bioinform..

[24] Kanehisa M, Furumichi M, Sato Y, Kawashima M, Ishiguro-Watanabe M (2023). KEGG for taxonomy-based analysis of pathways and genomes. Nucleic Acids Res..

[25] Yu G, Wang LG, Han Y, He QY (2012). clusterProfiler: An R package for comparing biological themes among gene clusters. Omics J. Integr. Biol..

[26] Lancaster SM, Sanghi A, Wu S, Snyder MP (2020). A customizable analysis flow in integrative multi-omics. Biomolecules.

[27] Huang ML, Hung YH, Lee WM, Li RK, Jiang BR (2014). SVM-RFE based feature selection and Taguchi parameters optimization for multiclass SVM classifier. Sci. World J..

[28] Dawkins JJ (2022). Gut metabolites predict *Clostridioides difficile* recurrence. Microbiome.

[29] Robin X (2011). pROC: An open-source package for R and S+ to analyze and compare ROC curves. BMC Bioinform..

[30] Buciuc M (2020). Utility of FDG-PET in diagnosis of Alzheimer-related TDP-43 proteinopathy. Neurology.

[31] Davatchi F (2014). The International Criteria for Behçet's Disease (ICBD): A collaborative study of 27 countries on the sensitivity and specificity of the new criteria. J. Eur. Acad. Dermatol. Venereol..

[32] Newman AM (2015). Robust enumeration of cell subsets from tissue expression profiles. Nat. Methods.

[33] Mukhopadhyay S (2019). Fibrinolysis and inflammation in venous thrombus resolution. Front. Immunol..

[34] Liederman Z, Chan N, Bhagirath V (2020). Current challenges in diagnosis of venous thromboembolism. J. Clin. Med..

[35] Wang J (2023). Current challenges in the prevention and management of post-thrombotic syndrome-towards improved prevention. Int. J. Hematol..

[36] Khoshbakht S, Başkurt D, Vural A, Vural S (2023). Behçet's disease: A comprehensive review on the role of HLA-B*51, antigen presentation, and inflammatory cascade. Int. J. Mol. Sci..

[37] Yang JY, Park MJ, Park S, Lee ES (2018). Increased senescent CD8+ T cells in the peripheral blood mononuclear cells of Behçet's disease patients. Arch. Dermatol. Res..

[38] Bochenek ML, Schütz E, Schäfer K (2016). Endothelial cell senescence and thrombosis: Ageing clots. Thromb. Res..

[39] Schmitt CA (2023). COVID-19 and cellular senescence. Nat. Rev. Immunol..

[40] Le Joncour A, Cacoub P, Boulaftali Y, Saadoun D (2023). Neutrophil, NETs and Behçet's disease: A review. Clin. Immunol. Orlando Fla..

[41] Yuan Y, Li X, Li M (2018). Overexpression of miR-17-5p protects against high glucose-induced endothelial cell injury by targeting E2F1-mediated suppression of autophagy and promotion of apoptosis. Int. J. Mol. Med..

[42] Fouad S, Hauton D, D'Angiolella V (2020). E2F1: Cause and consequence of DNA replication stress. Front. Mol. Biosci..

[43] Dong W, Zhan C (2022). Bioinformatic-based mechanism identification of E2F1-related ceRNA and E2F1 immunoassays in hepatocellular carcinoma. J. Gastrointest. Oncol..

[44] Gao L-N, Li Q, Xie J-Q, Yang W-X, You C-G (2021). Immunological analysis and differential genes screening of venous thromboembolism. Hereditas.

[45] Belghith M (2018). Cerebrospinal fluid IL-10 as an early stage discriminative marker between multiple sclerosis and neuro-Behçet disease. Cytokine.

[46] Kacem O, Kaabachi W, Dhifallah IB, Hamzaoui A, Hamzaoui K (2018). Elevated expression of TSLP and IL-33 in Behçet's disease skin lesions: IL-37 alleviate inflammatory effect of TSLP. Clin. Immunol..

[47] Ma L, Xue W, Ma X (2018). GATA3 is downregulated in osteosarcoma and facilitates EMT as well as migration through regulation of slug. OncoTargets Therapy.

[48] Truong V, Jain A, Anand-Srivastava MB, Srivastava AK (2021). Angiotensin II-induced histone deacetylase 5 phosphorylation, nuclear export, and Egr-1 expression are mediated by Akt pathway in A10 vascular smooth muscle cells. Am. J. Physiol. Heart Circ. Physiol..

[49] Chang S-F (2022). Blood reflux-induced epigenetic factors HDACs and DNMTs are associated with the development of human chronic venous disease. Int. J. Mol. Sci..

[50] Tsou PS (2016). Histone deacetylase 5 is overexpressed in scleroderma endothelial cells and impairs angiogenesis via repression of proangiogenic factors. Arthr. Rheumatol..

[51] Larsson P (2012). Role of histone acetylation in the stimulatory effect of valproic acid on vascular endothelial tissue-type plasminogen activator expression. PloS one.

[52] Pita-Thomas W, Mahar M, Joshi A, Gan D, Cavalli V (2019). HDAC5 promotes optic nerve regeneration by activating the mTOR pathway. Exp. Neurol..

[53] Kivanc D, Dasdemir S (2021). The relationship between defects in DNA repair genes and autoinflammatory diseases. Rheumatol. Int..

[54] Qiu W (2021). Analysis of the expression and prognostic value of MSH2 in pan-cancer based on bioinformatics. BioMed. Res. Int..

[55] Wolberg AS (2015). Venous thrombosis. Nat. Rev. Dis. Prim..

[56] Zhan H (2021). Novel insights into gene signatures and their correlation with immune infiltration of peripheral blood mononuclear cells in Behcet's disease. Front. Immunol..

[57] Hu D, Guan JL (2023). The roles of immune cells in Behçet's disease. Adv. Rheumatol. Lond. Engl..

[58] Abuduhalike R, Abudouwayiti A, Juan S, MaheMuti A (2023). Study on the mechanism of NLRP3/IL-1/ NF-κB signaling pathway and macrophage polarization in the occurrence and development of VTE. Ann. Vasc. Surg..

[59] Zhang Y (2021). Inflammasome activation promotes venous thrombosis through pyroptosis. Blood Adv..

[60] Desbois AC (2012). Immunosuppressants reduce venous thrombosis relapse in Behçet's disease. Arthr. Rheum..

