# Supplementary material for: Exploration of effective biomarkers for venous thrombosis embolism in Behçet’s disease based on comprehensive bioinformatics analysis
Source: Sci Rep. 2024 Jul 10;14:15884. doi: 10.1038/s41598-024-66973-3 (PMC11236978; doi:10.1038/s41598-024-66973-3)
Supplement: Supplementary file 1 — Supplementary Information. [file 41598_2024_66973_MOESM1_ESM.docx]

**Supplementary Material**

**Supplementary Table S1.** Clinical characteristics of the patients.

| Characteristics | BD without VTE | BD with VTE | *P* value |
| --- | --- | --- | --- |
|  | N=8 | N=6 |  |
| Age, years | 40.88±8.64 | 46.50±4.04 | 0.218 |
| Gender, n (%) |  |  | 1 |
| Male | 6(75.00) | 5(83.33) |  |
| Female | 2(25.00) | 1(16.67) |  |
| hs-CRP, mg/L | 3.77±1.99 | 10.26±3.27 | 0.0006 |
| ESR, mm/h | 11.38±5.66 | 25.5±9.63 | 0.0055 |
| Hemoglobin, g/L | 131.25±12.60 | 121.33±15.59 | 0.2119 |
| Leukocyte, 10^9^/L | 5.71±2.13 | 6.5±1.08 | 0.4218 |
| Platelets, 10^9^/L | 180.88±50.42 | 235.33±62.19 | 0.0949 |
| D-dimer, mg/L | 0.65±0.58 | 3.52±2.13 | 0.0204 |
| Homocysteine, μmol/L | 9.00±1.23 | 13.17±5.56 | 0.0597 |
| Smoking, n (%) | 1(12.50) | 1(16.67) | 1 |
| Hyperlipidemia, n (%) | 2(25.00) | 2(33.33) | 1 |

Abbreviations: hs-CRP, hypersensitive C-reactive protein; ESR, Erythrocyte Sedimentation Rate.

**Supplementary Table S2.** Primers used in the study.

| Gene | Forward primer | Reverse primer |
| --- | --- | --- |
| E2F1 | CAGAGCAGATGGTTATGG | CTGAAAGTTCTCCGAAGA |
| GATA3 | GCCCCTCATTAAGCCCAAG | TTGTGGTGGTCTGACAGTTCG |
| HDAC5 | TCTTGTCGAAGTCAAAGGAGC | GAGGGGAACTCTGGTCCAAAG |
| MSH2 | AGGCATCCAAGGAGAATGATTG | GGAATCCACATACCCAACTCCAA |
| β-actin | CATGTACGTTGCTATCCAGGC | CTCCTTAATGTCACGCACGAT |

**Supplementary Table S3.** Functional enrichment analysis of BD-related DEGs in VTE.

| Description | Count | Gene |
| --- | --- | --- |
| **Biological Process** |  |  |
| leukocyte migration | 12 | STK39/S1PR1/SWAP70/GATA3/FUT7/CNR2/GYPA/ESAM/MAPK14/ATP1B2/SELP/GYPB |
| nerve growth factor processing | 2 | NGF/FURIN |
| embryonic hemopoiesis | 3 | GATA3/TAL1/PBX1 |
| mast cell differentiation | 2 | GATA3/TAL1 |
| myotube differentiation | 5 | CACNA1H/HDAC5/WNT10B/MAPK14/XK |
| xenobiotic transport | 2 | SLC2A1/LRRC8A |
| viral life cycle | 8 | WWP1/NDC1/NUP160/RAB1B/INPP5K/AAAS/GYPA/FURIN |
| negative regulation of cellular metabolic process | 27 | HMG20A/SWAP70/MSH2/CDKN1B/WWP1/GATA3/AEBP2/DNAJC10/E2F1/QSOX1/HDAC5/BRMS1/RARG/POLDIP2/NGF/SNTA1/WNT10B/CIC/INPP5K/AP2A1/PI16/TAL1/DLL4/MAPK14/NECAB2/FURIN/SIAH2 |
| regulation of biological quality | 36 | STK39/S1PR1/SWAP70/EVL/DOCK10/GATA3/SMC5/DNAJC10/E2F1/SLC2A1/CACNA1H/QSOX1/RARG/NGF/HRH2/ECE1/SNTA1/WNT10B/AGTRAP/INPP5K/KCNH2/LRRC8A/TBXA2R/CNR2/GRASP/AVPR1B/HBE1/TAL1/TGM2/MAPK14/ATP1B2/SPR/FURIN/SELP/RILP/XK |
| peptide hormone processing | 3 | NGF/ECE1/FURIN |
| **Cellular Component** |  |  |
| endomembrane system | 40 | S1PR1/CDKN1B/SARAF/MPHOSPH9/DNAJC10/NDC1/NUP160/SLC2A1/QSOX1/HDAC5/RAB1B/AEN/PIGQ/MINK1/NGF/ECE1/GJB1/AGTRAP/INPP5K/ABHD4/DOK3/AP2A1/SLC6A17/LRP3/FUT7/FKBP10/MBOAT2/TBXA2R/AAAS/AVPR1B/TGM2/MAPK14/ROGDI/CCDC3/FURIN/ACRBP/GNAZ/SELP/RILP/SIAH2 |
| plasma membrane part | 25 | STK39/S1PR1/CA11/SLC2A1/CACNA1H/PTGIR/MINK1/ECE1/GJB1/SNTA1/INPP5K/KCNH2/LRRC8A/AP2A1/SLC6A17/TBXA2R/CNR2/GRASP/TGM2/ATP1B2/ROGDI/STAC2/CEACAM4/SELP/GYPB |
| nucleoplasm | 31 | STK39/S1PR1/DOCK10/HMGN1/MSH2/DBR1/CDKN1B/GATA3/ILKAP/SCAF8/AEBP2/SMC5/ZCCHC8/E2F1/HDAC5/BRMS1/RARG/AEN/CIC/AGTRAP/TBXA2R/AAAS/MYBL2/EPOP/UBE2M/TAL1/PBX1/MAPK14/SPR/CDC34/SIAH2 |
| ESC/E(Z) complex | 2 | AEBP2/EPOP |
| nuclear pore | 3 | NDC1/NUP160/AAAS |
| host | 3 | NDC1/NUP160/AAAS |
| host cell | 3 | NDC1/NUP160/AAAS |
| intrinsic component of plasma membrane | 14 | S1PR1/SLC2A1/CACNA1H/PTGIR/KCNH2/LRRC8A/SLC6A17/TBXA2R/CNR2/TGM2/ATP1B2/CEACAM4/SELP/GYPB |
| other organism | 3 | NDC1/NUP160/AAAS |
| other organism cell | 3 | NDC1/NUP160/AAAS |
| **Molecular Function** |  |  |
| enzyme binding | 22 | STK39/DOCK10/MSH2/CDKN1B/SCAF8/DNAJC10/E2F1/SLC2A1/HDAC5/PTGIR/SNTA1/KCNH2/AP2A1/TBXA2R/RAB3IL1/GRASP/AVPR1B/TAL1/MAPK14/ATP1B2/FURIN/RILP |
| structural constituent of nuclear pore | 2 | NDC1/NUP160 |
| sphingolipid binding | 2 | S1PR1/SELP |
| ATPase activator activity | 2 | DNAJC10/ATP1B2 |
| ATPase binding | 3 | DNAJC10/SNTA1/ATP1B2 |
| kinase binding | 9 | STK39/MSH2/CDKN1B/E2F1/SLC2A1/HDAC5/AP2A1/AVPR1B/MAPK14 |
| ATPase regulator activity | 2 | DNAJC10/ATP1B2 |
| proximal promoter sequence-specific DNA binding | 7 | GATA3/AEBP2/E2F1/HDAC5/MYBL2/TAL1/PBX1 |
| protein kinase binding | 8 | STK39/MSH2/CDKN1B/E2F1/HDAC5/AP2A1/AVPR1B/MAPK14 |
| cannabinoid receptor activity | 1 | CNR2 |
| **KEGG** |  |  |
| Cushing syndrome | 5 | CDKN1B/E2F1/CACNA1H/WNT10B/PBX1 |
| Endocrine resistance | 4 | CDKN1B/E2F1/DLL4/MAPK14 |
| Malaria | 3 | GYPA/SELP/GYPB |
| Neuroactive ligand-receptor interaction | 6 | S1PR1/PTGIR/HRH2/TBXA2R/CNR2/AVPR1B |
| Cellular senescence | 4 | E2F1/HIPK4/MYBL2/MAPK14 |
| Th1 and Th2 cell differentiation | 3 | GATA3/DLL4/MAPK14 |
| Calcium signaling pathway | 4 | CACNA1H/HRH2/TBXA2R/AVPR1B |
| Platelet activation | 3 | PTGIR/TBXA2R/MAPK14 |
| Endocrine and other factor-regulated calcium reabsorption | 2 | AP2A1/ATP1B2 |
| FoxO signaling pathway | 3 | S1PR1/CDKN1B/MAPK14 |

# Abbreviations: BD, Behçet's disease; VTE, Venous thrombosis embolism; DEG, differentially expressed genes; KEGG, kyoto encyclopedia of genes and genomes.

**Supplementary Table S4.** Complete list of DEGs from three algorithms via CytoHubba plug-in.

| Degree | Betweenness | Closeness | Intersection |
| --- | --- | --- | --- |
| GATA3 | GATA3 | GATA3 | GATA3 |
| TAL1 | CDKN1B | MAPK14 | CDKN1B |
| GYPB | NGF | TAL1 | NGF |
| MAPK14 | MAPK14 | CDKN1B | MAPK14 |
| GYPA | SELP | NGF | SELP |
| NGF | TAL1 | GYPA | TAL1 |
| CDKN1B | SLC2A1 | SLC2A1 | SLC2A1 |
| SELP | CDC34 | FUT7 | CDC34 |
| FECH | FUT7 | SELP | FUT7 |
| HBE1 | E2F1 | GYPB | E2F1 |
| FUT7 | GYPA | PBX1 | GYPA |
| HDAC5 | TBXA2R | HDAC5 | TBXA2R |
| RARG | AP2A1 | RARG | AP2A1 |
| CDC34 | HDAC5 | HBE1 | HDAC5 |
| PBX1 | DNAJC10 | E2F1 | DNAJC10 |
| SLC2A1 | MSH2 | MYBL2 | MSH2 |
| E2F1 | FURIN | CDC34 | FURIN |
| MYBL2 | GYPB | DLL4 | GYPB |
| TBXA2R | MYBL2 | FECH | MYBL2 |
| AP2A1 | RARG | AP2A1 | RARG |
| KCNH2 | FECH | TBXA2R | FECH |
| DNAJC10 | HBE1 | FURIN | HBE1 |
| AAAS | PBX1 | ILKAP | PBX1 |
| NUP160 | KCNH2 | STK39 |  |
| MSH2 | FKBP10 | MSH2 |  |
| FURIN | RUNDC3A | XK |  |
| NDC1 | TGM2 | ESAM |  |
| XK | PRRX2 | DNAJC10 |  |
| FKBP10 | ATP1B2 | BRMS1 |  |
| RUNDC3A | PIGQ | UBE2M |  |

Abbreviations: DEG, differentially expressed genes.

**Supplementary Table S5 Therapeutic agents screening**

| Term | P-value | Genes |
| --- | --- | --- |
| CHEMBL35349 CTD 00001274 | 2.45E-05 | MSH2; E2F1 |
| chlorpromazine CTD 00005648 | 1.88E-04 | MSH2; E2F1 |
| 2-Nonenal, 4-hydroxy-, (2E,4R)- CTD 00001295 | 0.001101815 | E2F1; GATA3 |
| Dronabinol CTD 00006853 | 0.002354101 | E2F1; GATA3 |
| N'-Nitrosonornicotine CTD 00000423 | 0.002597617 | GATA3 |
| Trabectedin CTD 00003298 | 0.002597617 | E2F1 |
| Vorinostat CTD 00003560 | 0.002627457 | HDAC5; GATA3 |
| carmustine CTD 00005595 | 0.002676484 | MSH2; E2F1 |
| COUMESTROL CTD 00005717 | 0.002768661 | MSH2; E2F1; GATA3 |
| N-Methyl-N-nitrosourea CTD 00006319 | 0.003196351 | MSH2 |


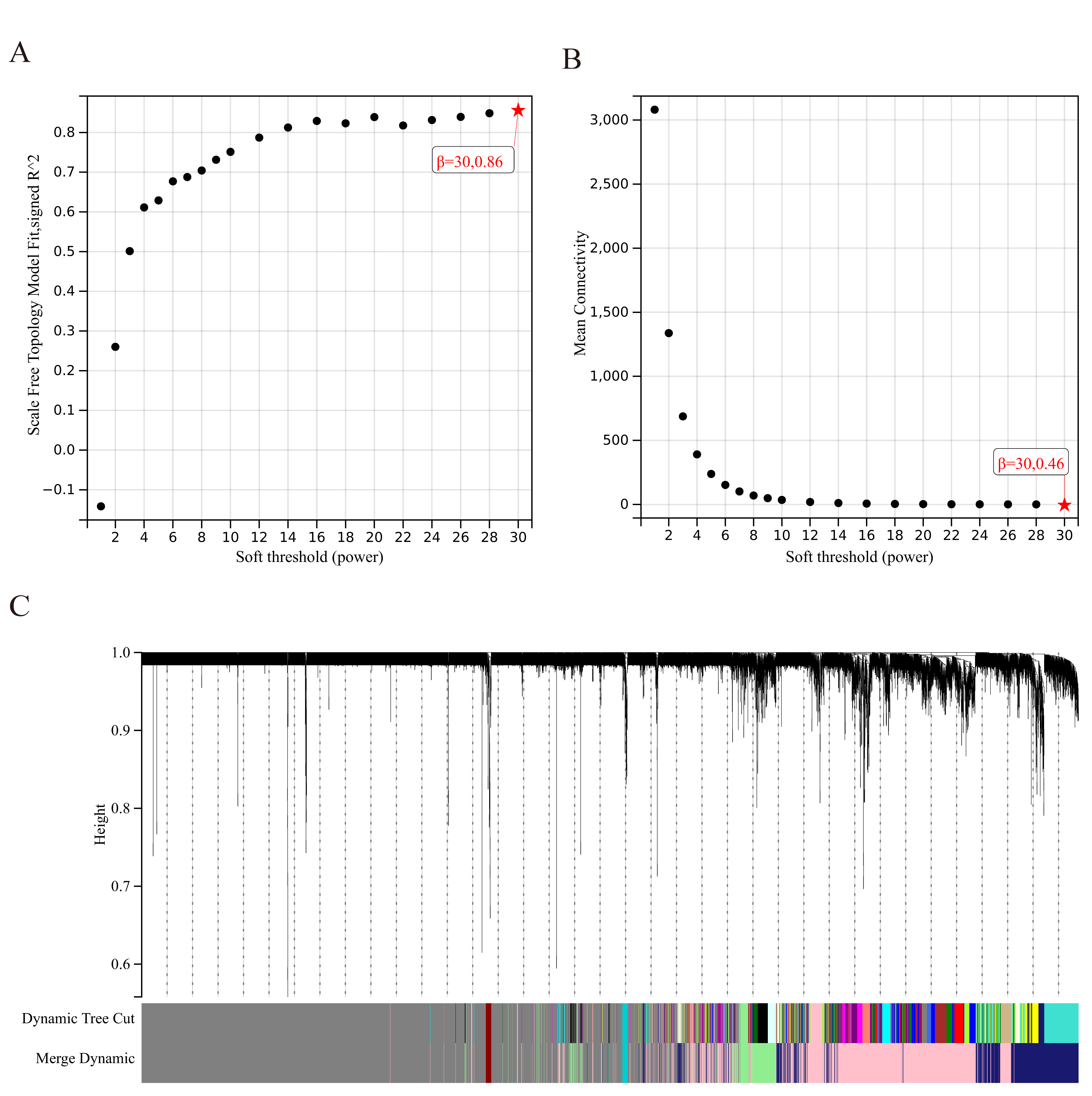


**Supplementary Figure S1.** Soft thereshold selection and gene cluster tree via WGCNA of BD

(A-B) The soft threshold selection. β=30 was choose as the most appropriate threshold.

(C) Gene cluster tree of different modules.

Abbreviations: WGCNA, weighted gene co-expression network analysis; BD, Behçet's disease.


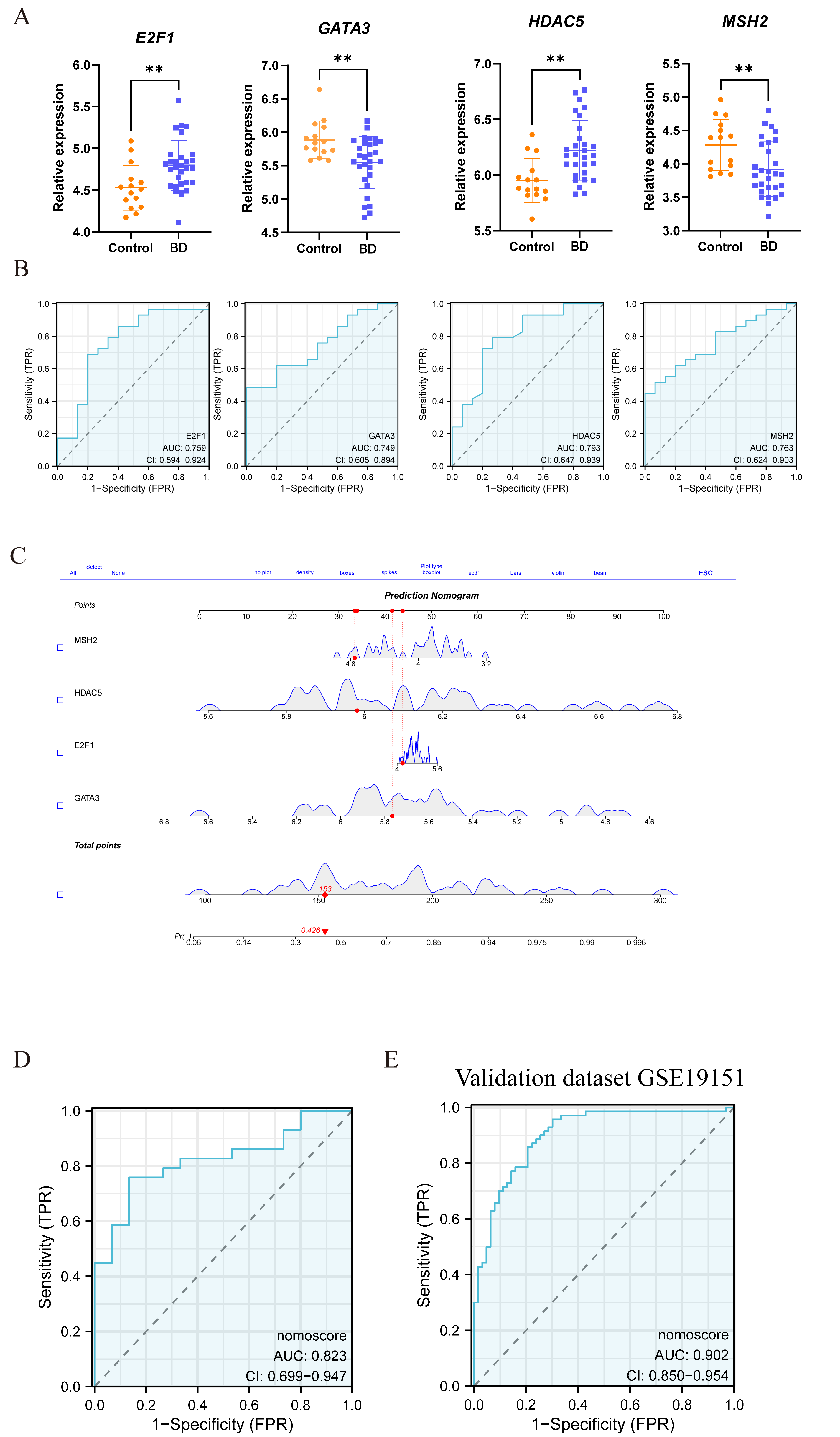


**Supplementary Figure S2.** Evaluation of ROC and nomogram construction in

BD and the clinical diagnostic value of the nomogram in the validation dataset.

(A) Differential expression is observed in four genes when comparing the and control groups (**, p < 0.01).

(B) The ROC curve was utilized to evaluate the diagnostic efficacy of the four hub genes (E2F1, GATA3, HDAC5, and MSH2) in identifying BD, demonstrating satisfactory diagnostic performance with AUC and 95% CI displayed in each panel.

(C) The construction of a diagnostic nomogram employing the four genes aims to enhance the diagnosis of BD.

(D) The ROC curve for the nomogram is depicted in patients with BD.

(E) The diagnostic performance of the nomogram in VTE with BD from the ROC curve.

Abbreviations: BD, Behçet's disease; VTE, Venous thrombosis embolism; ROC, receiver operating characteristic curve. AUC, area under the curve; CI, confidence interval.


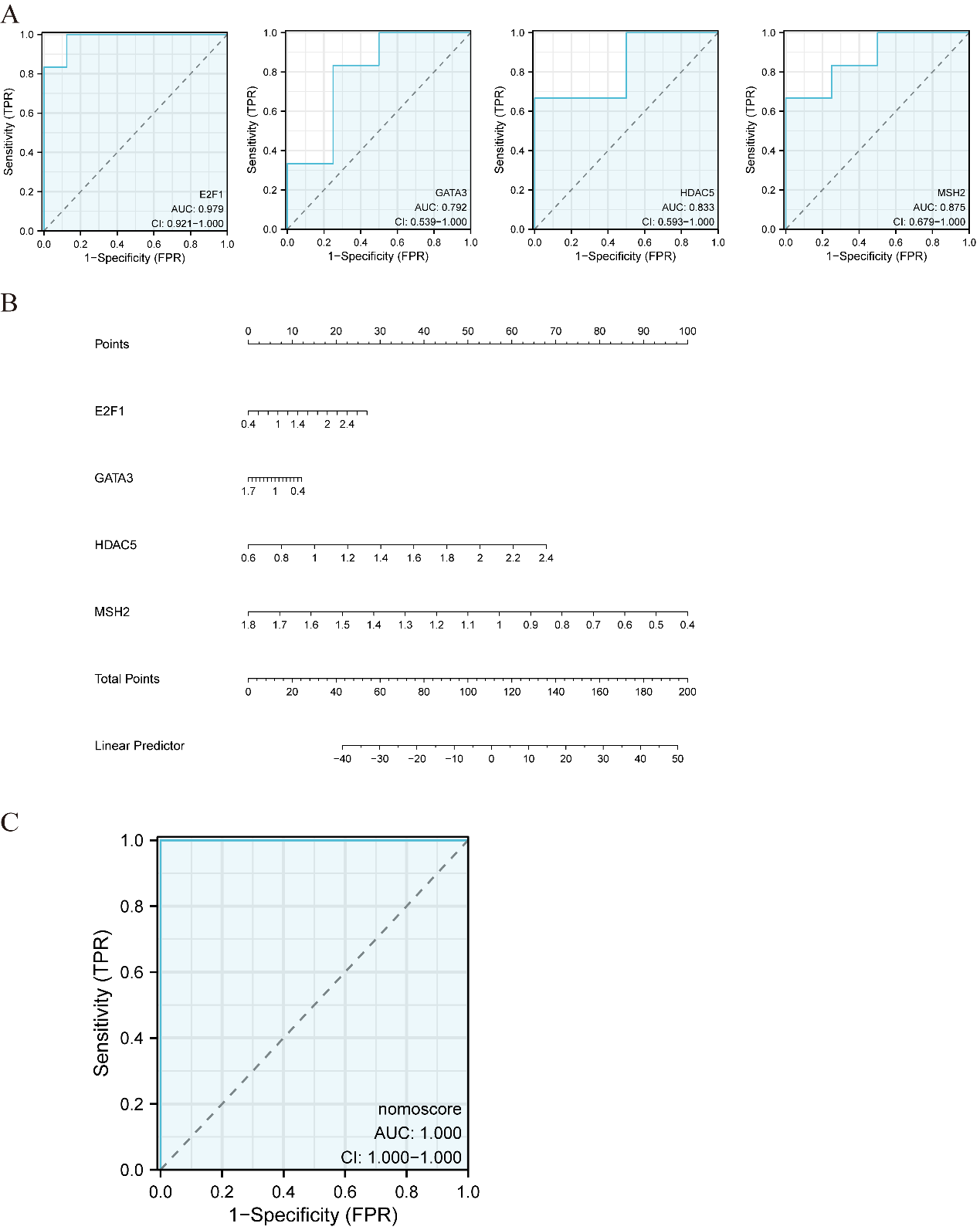


**Supplementary Figure S3.** Validation of expression of three hub genes by RT-qPCR and nomogram construction.

(A) The ROC curve was utilized to evaluate the diagnostic efficacy of the four hub genes (E2F1, GATA3, HDAC5, and MSH2) in identifying VTE in patients with BD, demonstrating satisfactory diagnostic performance with AUC and 95% CI displayed in each panel.

(B) A nomogram was developed using four externally validated DEGs. Each DEG corresponds to a score on the nomogram. The final score was determined by adding the scores for each DEG.

(C) ROC curve of the nomogram revealed DEGs with prominent predictive value for VTE in patients with BD.


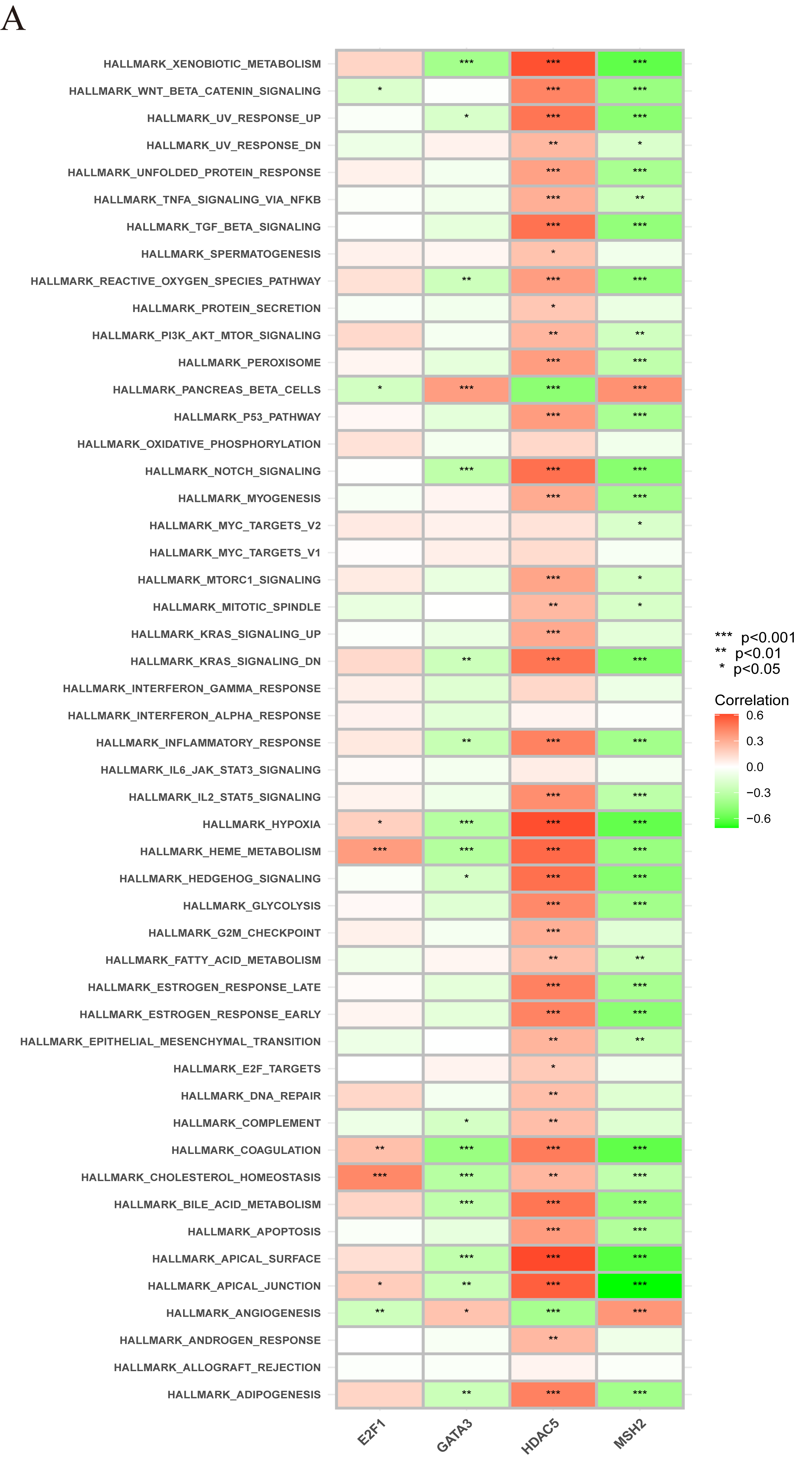


**Supplementary Figure S4.** Correlation analysis of the three candidate biomarkers with hallmark gene sets via ssGSEA

Abbreviations: ssGSEA, Single-sample gene-set enrichment analysis.


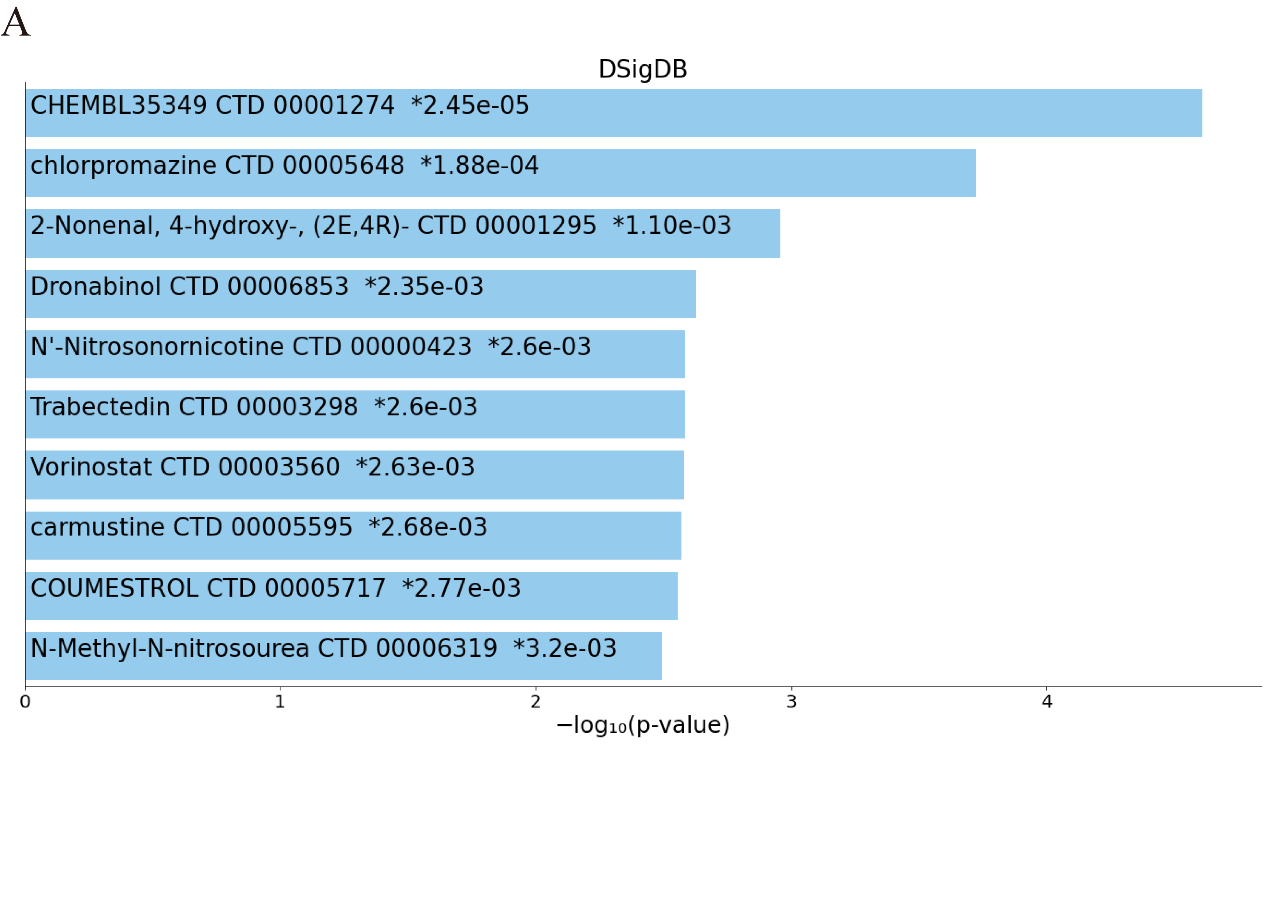


**Supplementary Figure S5.** Therapeutic agent screening

(A) Bar chart of top enriched terms from the DSigDB gene set library. The top 10 enriched terms for the input gene set are displayed based on the -log10(p-value), with the actual p-value shown next to each term. The term at the top has the most significant overlap with the input query gene set.
